# DeepNitro: Prediction of Protein Nitration and Nitrosylation Sites by Deep Learning

**DOI:** 10.1016/j.gpb.2018.04.007

**Published:** 2018-09-27

**Authors:** Yubin Xie, Xiaotong Luo, Yupeng Li, Li Chen, Wenbin Ma, Junjiu Huang, Jun Cui, Yong Zhao, Yu Xue, Zhixiang Zuo, Jian Ren

**Affiliations:** 1 State Key Laboratory of Oncology in South China, Cancer Center, Collaborative Innovation Center for Cancer Medicine, School of Life Sciences, Sun Yat-sen University, Guangzhou 510060, China; 2 Department of Bioinformatics & Systems Biology, MOE Key Laboratory of Molecular Biophysics, College of Life Science and Technology and the Collaborative Innovation Center for Biomedical Engineering, Huazhong University of Science and Technology, Wuhan 430074, China

**Keywords:** Protein nitration and nitrosylation, Deep learning, Web service, Functional site prediction, Feature extraction

## Abstract

**Protein nitration and nitrosylation** are essential post-translational modifications (PTMs) involved in many fundamental cellular processes. Recent studies have revealed that excessive levels of nitration and nitrosylation in some critical proteins are linked to numerous chronic diseases. Therefore, the identification of substrates that undergo such modifications in a site-specific manner is an important research topic in the community and will provide candidates for targeted therapy. In this study, we aimed to develop a computational tool for predicting nitration and nitrosylation sites in proteins. We first constructed four types of encoding features, including positional amino acid distributions, sequence contextual dependencies, physicochemical properties, and position-specific scoring features, to represent the modified residues. Based on these encoding features, we established a predictor called DeepNitro using **deep learning** methods for predicting protein nitration and nitrosylation. Using *n*-fold cross-validation, our evaluation shows great AUC values for DeepNitro, 0.65 for tyrosine nitration, 0.80 for tryptophan nitration, and 0.70 for cysteine nitrosylation, respectively, demonstrating the robustness and reliability of our tool. Also, when tested in the independent dataset, DeepNitro is substantially superior to other similar tools with a 7%−42% improvement in the prediction performance. Taken together, the application of deep learning method and novel encoding schemes, especially the position-specific scoring feature, greatly improves the accuracy of nitration and nitrosylation site prediction and may facilitate the prediction of other PTM sites. DeepNitro is implemented in JAVA and PHP and is freely available for academic research at http://deepnitro.renlab.org.

## Introduction

Proteins undergo various post-translational modifications (PTMs) to maintain their functions. Of the nearly 460 types of PTMs, protein nitration and nitrosylation are special epigenetic regulations that are mainly caused by unfavorable side reactions from metabolic processes during normal cellular activities. Under physiological conditions, nitric oxide (NO) is produced by NO synthases (NOS) [1] and subsequently diffuses through cell membranes. As a signaling molecule, NO is able to regulate many vascular and neuronal signaling pathways, as well as mitochondrial proliferation [2], [3]. In the presence of oxidants, NO can be further converted into different reactive nitrogen species (RNS), such as nitrous acid (HNO_2_), dinitrogen trioxide (N_2_O_3_), nitrosyl anion (NO^−^), nitrosyl cation (NO^+^), and nitrogen dioxide radical (NO_2_) [4]. These RNS are able to induce nitration of proteins, thereby changing their chemical properties and reforming their tertiary structures. It has been reported that nitration can occur at amino acid residues such as tyrosine [5] and tryptophan [6]. In addition to protein nitration, RNS can also lead to the formation of protein *S*-nitrosylation by covalently attaching the nitrosyl group to the sulfur atom of cysteine [7].

The addition of nitro or nitrosyl groups to certain amino acid residues is an important mechanism for regulating the biological functions of specific cellular proteins by conferring particular physicochemical properties to the modified residues [8]. Recent reports have demonstrated that tyrosine nitration, tryptophan nitration, and *S*-nitrosylation play critical roles in multiple physiological and pathological processes, including cell signaling [9], immune response [10], cell death [11], transcriptional regulation [12], and protein activity [13]. The abnormal abundance of these modifications may lead to deleterious consequences. Many chronic diseases such as diabetes [14], atherosclerosis [15], chronic renal failure [16], cardiovascular diseases [17], and neurological disorders [18] are evidently linked to the aforementioned modifications. Therefore, the identification of substrates that undergo nitration or *S*-nitrosylation in a site-specific manner is important for providing potential guidance for the development of new therapeutic strategies and drugs.

At present, the large-scale identification of nitration or *S*-nitrosylation sites mainly relies on mass spectrometry-based methods [19]. However, because the level of endogenously nitrated or nitrosylated proteins in the cell is usually very low, highly efficient *in vivo* detection of individual nitrated or nitrosylated proteins has long remained a major methodological issue. Prior immunoprecipitation with specific antibodies is helpful to improve efficiency, but the immunoprecipitation step usually requires complicated procedures, resulting in a laborious, inefficient, and expensive process. In this regard, further efforts are needed to improve the efficiency of current proteomic methodologies so that they can be applied in more research cases and facilitate the investigation of RNS-induced protein modifications.

In contrast to the time-consuming and expensive experimental methods, computational approaches for discovering PTM sites have attracted considerable attention because of their convenience and efficiency. To date, several programs have been developed for predicting the nitration and *S*-nitrosylation site, such as GPS-YNO2 [20] and iNitro-tyr [21] for tyrosine nitration, as well as GPS-SNO [22], iSNO-PseAAc [23], and SNOSite [24] for *S*-nitrosylation site prediction. However, many issues remain in these algorithms, leaving a lot of room for improvement. First, the existing tools generally rely on traditional shallow machine learning methods for prediction, which fail to learn the underlying biological features of protein modifications lacking a consensus sequence, such as nitration or nitrosylation, thus leading to inaccurate predictions of potential modification sites. Second, feature selection is critical for machine learning-based algorithms. However, the current feature extraction methods used in the methods above are unable to fully characterize the biological properties of the potential sites, resulting in disappointing performance. Third, up to now, there are no available tools for the prediction of tryptophan nitration, which limit the comprehensive study of protein nitration and nitrosylation in mammalian. Therefore, the development of a novel tool that is able to extract high-level features from the input sequences and produce reliable prediction results with sufficient accuracy remains an important problem to be solved.

Recently, the application of deep learning algorithm in machine learning research has attracted more and more attention. By learning a suitable representation of the input data, the raw vectors can be transformed into highly abstract features through propagating the whole model. Theoretically, superposition of sufficient level of neural network can increase the ability of feature extraction, resulting in more accurate interpretation of data. However, the so-called gradient diffusion problem has greatly restricted the depth of neural network model [25]. Until 2006, Hinton et al. proposed the layer-wise training strategy [26] to solve this problem properly and the deep neural network turned to practical account. Nowadays, many new techniques have been developed for the training of deep learning model, including Rectified Linear Unit (ReLU) [27], dropout training [28], regularization [29], and momentum method [30]. With these advantages, deep learning has achieved state-of-the-art accuracy on many prediction tasks, such as image classification [31], speech recognition [32], and natural language processing [33]. Inspired by its excellent performance, deep learning is most recently applied in research field of computational biology. Typical applications include transcript factor binding site detection [34], protein structure prediction [35], RNA splicing prediction [36], and pathogenic variant identification [37]. In view of this, introducing deep learning algorithm into the prediction of nitration and nitrosylation sites would be a promising move to solve the current deficiencies in learning of in-depth biological features and provide more meaningful candidates for further experimental considerations.

In this work, by applying the deep learning algorithm together with effective feature extraction methods, we developed a novel software called DeepNitro to predict protein nitration and nitrosylation sites. For convenience, a webserver has also been developed and is freely available at http://deepnitro.renlab.org.

## Methods

### Preparation of the dataset

To collect the training dataset, we first searched the scientific literature published before Jun 30th, 2015 from PubMed using the keywords “Nitration”, “Nitrated”, ”Nitrosylation”, or “Nitrosylated”. By manually reading the retrieved articles, we collected the exact locations of all the experimentally-verified nitration and nitrosylation sites. After removing redundant sites, we collected a total of 1518 tyrosine nitration sites, 66 tryptophan nitration sites, and 4762 *S*-nitrosylation sites in 3113 unique proteins. As previously described [20], [22], we treated the modified residues (Y for tyrosine nitration, W for tryptophan nitration, and C for *S*-nitrosylation) that have been reported in the published literature as positive data. Accordingly, the same types of non-modified residues present in the same sequence were considered as negative data. Generally speaking, if the modified residues included in the training dataset are redundant with too many homologous sites, the training process will carry a significant risk of model overfitting, leading to spurious prediction. To avoid this possibility, we first clustered the collected protein sequences using CD-Hit with an identity threshold of 40%. For proteins clustered in the same homologous group, we re-aligned them using the Smith-Waterman algorithm and checked the results manually. If two given positive sites or negative sites shared an identical flanking sequence around the modified residues, only one item was reserved for the model training. Finally, for tyrosine nitration, we constructed a training set of 1210 positive and 8043 negative sites. For tryptophan nitration, the training data contained 66 positive sites and 155 negative sites. For *S*-nitrosylation, we retained 3409 positive sites and 17,453 negative sites as the training set (Table S2).

Particularly, to evaluate the actual performance of our prediction model and compare it with other existing tools, we also constructed the independent test set for tyrosine nitration and *S*-nitrosylation. To ensure a fair comparison, the independent test set does not contain any nitration or nitrosylation sites that are present in the training set of previously-published tools. To meet this criterion, we constructed the test set by focusing on the most recently collected data and removing duplicate sites that had been used in training sets of other tools. In total, 189 positive sites and 1182 negative sites were selected for the independent test set for tyrosine nitration. For the independent test set for *S*-nitrosylation, another 485 positive sites and 4947 negative sites were included (Table S2). However, due to the limited data availability, an additional test set for tryptophan nitration was not constructed.

### Feature encoding scheme

Before constructing the prediction model for protein nitration and nitrosylation, we first transferred the known modification sites into a set of feature vectors that can be directly recognized by classification algorithms. As presented in our previously published papers [20], [22], the chemical properties and amino acid composition around the modified residue may affect the specificity of nitration and nitrosylation. Therefore, we constructed the encoding scheme by extracting sequence or biochemical features from the flanking sequence of length *L* in which the modified or non-modified site is located at the central position. Here, we defined the aforementioned region as a nitration or nitrosylation peptide. Specifically, the optimal window size *L* can be selected by the subsequent 4-fold cross-validation. A detailed description of the encoding scheme is provided below.

#### One-hot encoding of the nitration or nitrosylation peptide

To precisely describe the amino acid distribution at each position in the nitration or nitrosylation peptide, we transferred the 20 natural amino acid residues and a gap character filling the sequence termini into a 21-dimensional binary array. Accordingly, one-hot encoding for a peptide with a window size of *L* would result in a binary feature vector of *L* × 21 dimensions.

#### The physical and chemical properties of the nitration or nitrosylation peptide

The physiochemical environment is responsible for the formation of a covalent linkage between proteins and nitrogenous groups [38], [39]. Thus, measurement of the physiochemical properties of the candidate residues may be particularly important for the accurate prediction of nitration and nitrosylation sites. Therefore, we introduced the property factor representation (PFR) [40] in our work and encoded the flanking regions into a set of physiochemical features. Using the encoding table (Table S3) from PFR, a given amino acid residue *X* can be encoded into a physiochemical feature vector *x* of dimensionality in 10 as defined below.

(1) $$x=\left(f_{x}^{1}\text{,}f_{x}^{2}\text{,}f_{x}^{3}\text{,}\cdots \text{,}f_{x}^{10}\right)$$

where $f_{x}^{i}$ denotes the *i-*th property factor for amino acid residue *x*. Accordingly, a nitration or nitrosylation peptide with length of *L* can be represented as a 10 × *L* dimensional numeric vector. Similar to one-hot encoding, the gap characters filling the sequence termini are encoded with vectors of zeros.

#### *k*-Space spectrum encoding

To depict the sequence context of a nitration or nitrosylation peptide, we calculated the *k*-space spectrum in our encoding scheme. When denoting a flanking region, we first scanned through the whole sequence with a sliding window of length *k* and counted the number of all possible amino acid pairs (*e.g.*, LxxxE is a three-space amino acid pair) for subsequent use. Specifically, for peptide with gap characters, we simply ignored the gapped sequence and encoded *k*-space spectrum only in meaningful regions. We represented a specific amino acid pair as *A_i_A_j_*. A total of 400 amino acid pairs can be obtained for any given protein sequence. Then, the frequency of a *k*-space amino acid pair was calculated as follows.

(2) $$f_{i,j}=\frac{A_{i}A_{j}}{L-k+1}$$

And the amino acid spectrum can be constructed by calculating the frequency of all possible amino acid pairs.

(3) $$\text{spectrum}={\left(f_{1,1}\text{,}f_{1,2}\text{,}f_{1,3}\text{,}\cdots \text{,}f_{10,10}\text{,}\cdots \text{,}f_{20,20}\right)}_{400}$$

The *k* values can be adjusted in a range of 0–4 to capture more information about the sequence dependency. Finally we obtained a 2000 dimensional vector for the amino acid spectrum.

#### Encoding with the position-specific scoring matrix

To depict the sequence conservation of nitration or nitrosylation sites, we developed the following position-specific scoring matrix (PSSM) encoding scheme in our prediction algorithm. Based on the model proposed by Vacic et al. [41], we first calculated the statistical significance of the differences in the frequencies of symbol occurrence between the positive and negative datasets. Let *P* and *Q* be the positive and negative samples, and *|P|* and *|Q|* be the numbers of sequences in the corresponding sample, respectively; and *P_i_* be the *i-*th peptide in the positive dataset and *P_ij_* be the *j*th position in peptide *P_i_*. For each position in *P* and for each symbol *a* from the alphabet (including 20 natural amino acids and a gap character), a binary vector $X_{p}^{j,a}$can be generated as shown below.

(4) $$X_{p}^{j,a}=\left(I_{1}\text{,}I_{2}\text{,}\cdots \text{,}I_{\left|p\right|}\right)$$

where *I_i_* can be calculated as follows.

(5) $$I_{i}=\left\{\begin{array}{cc} 1 & P_{\mathrm{ij}}=a\\ 0 & P_{\mathrm{ij}}\neq a \end{array}\right)$$

The vector $X_{Q}^{j,a}$ for the negative sample can be generated in a similar way. Then, we constructed a null hypothesis that vectors $X_{p}^{j,a}$ and $X_{Q}^{j,a}$ were sampled from the same distribution, or, in other words, the occurrence probabilities for amino acid *a* are identical at position *j* in both samples. According to this hypothesis, we could compute the *P* value using a two-sample *t*-test [41]. However, since nitration and nitrosylation can only occur in specific amino acid residues, the central position in both the positive and negative data set would be the same. Therefore, when computing *P* value in such case, the variance in vectors $X_{p}^{j,a}$ and $X_{Q}^{j,a}$ would be zero, thereby making the *t* statistics incalculable. To fix this issue, the central position of the input peptide should be discarded. Accordingly, the significant PSSM *P* values are constructed as shown below,

(6) $$P_{\text{PSSM}}\;\text{=}\;\left[\begin{array}{cccccc} p_{\text{1,1}} & \cdots & {\text{p}}_{\text{1,k - 1}} & {\text{p}}_{\text{1,k + 1}} & \cdots & {\text{p}}_{\text{1,L}}\\ {\text{p}}_{\text{2,1}} & \cdots & {\text{p}}_{\text{2,k - 1}} & {\text{p}}_{\text{2,k + 1}} & \cdots & {\text{p}}_{\text{2,L}}\\ \vdots & \vdots & \vdots & \vdots & \vdots & \vdots \\ {\text{p}}_{\text{21,1}} & \cdots & {\text{p}}_{\text{21,k - 1}} & {\text{p}}_{\text{21,k + 1}} & \cdots & {\text{p}}_{\text{21,L}} \end{array}\right]$$

where *L* is the length of the flanking region, *p_i,j_* denotes the *P* value of the *i*th amino acid in the *j*th position for a given set of nitration or nitrosylation peptide, and *k* refers to the central position. Notably, for the peptide sequence, the matrix above would be in a dimension of 21 × (*L* − 1).

Although the significant PSSM can represent the differences in sequence conservation between positive and negative sites, the positive and negative tendency, unfortunately, cannot be inferred from such a matrix. To address this issue, we further established the following computational process to measure the conservation tendency of a given set of nitration or nitrosylation peptide. Specifically, we first calculated the observed frequency of an amino acid *a* at position *j* and constructed a frequency PSSM as shown below,

(7) $${\text{F}}_{\text{Pos}}\;\text{=}\;\left[\begin{array}{cccccc} {\text{f}}_{\text{1,1}} & \cdots & {\text{f}}_{\text{1,k - 1}} & {\text{f}}_{\text{1,k + 1}} & \cdots & {\text{f}}_{\text{1,L}}\\ {\text{f}}_{\text{2,1}} & \cdots & {\text{f}}_{\text{2,k - 1}} & {\text{f}}_{\text{2,k + 1}} & \cdots & {\text{f}}_{\text{2,L}}\\ \vdots & \vdots & \vdots & \vdots & \vdots & \vdots \\ {\text{f}}_{\text{21,1}} & \cdots & {\text{f}}_{\text{21,k - 1}} & {\text{f}}_{\text{21,k + 1}} & \cdots & {\text{f}}_{\text{21,L}} \end{array}\right]$$

where *F_Pos_* denotes the frequency PSSM for the positive peptides, and *f_i,j_* represents the observed frequency of the *i-*th symbol from the alphabet in the *j-*th position. Accordingly, the frequency PSSM for negative peptides (denoted as *F_Neg_*) can also be calculated following the same approach. Based on *F_Pos_* and *F_Neg_*, we next computed the final encoding PSSM as shown below,

(8) $${\text{E}}_{\text{PSSM}}\;\text{=}\;\left[\begin{array}{cccccc} {\text{E}}_{\text{1,1}} & \cdots & {\text{E}}_{\text{1,k - 1}} & {\text{E}}_{\text{1,k + 1}} & \cdots & {\text{E}}_{\text{1,L}}\\ {\text{E}}_{\text{2,1}} & \cdots & {\text{E}}_{\text{2,k - 1}} & {\text{E}}_{\text{2,k + 1}} & \cdots & {\text{E}}_{\text{2,L}}\\ \vdots & \vdots & \vdots & \vdots & \vdots & \vdots \\ {\text{E}}_{\text{21,1}} & \cdots & {\text{E}}_{\text{21,k - 1}} & {\text{E}}_{\text{21,k + 1}} & \cdots & {\text{E}}_{\text{21,L}} \end{array}\right]$$

where *E_i,j_* is calculated as below.

(9) $$\delta_{i,j}=\frac{f_{i,j}^{\mathrm{Pos}}-f_{i,j}^{\mathrm{Neg}}}{p_{i,j}}$$

(10) $${\text{E}}_{i,j}=\left\{\begin{array}{cc} \text{ln}\left(\left|\delta_{i,j}\right|+1\right) & \delta_{i,j}⩾0\\ \text{ln}\left(\left|\delta_{i,j}\right|+1\right) & \delta_{i,j}<0 \end{array}\right)$$

The final encoding PSSM represents the conservation tendency of the nitration or nitrosylation peptides. If *E_i,j_* is greater than zero, then the amino acid at this position is more likely to be observed in the positive sites; conversely, the amino acid would have a better chance of appearing in the negative sites. Using *E_PSSM_* (Table S4), we encode a nitration or nitrosylation peptide into a numeric vector of dimension *L* − 1.

### Deep neural network for predicting potential nitration or nitrosylation sites

**Figure 1:**
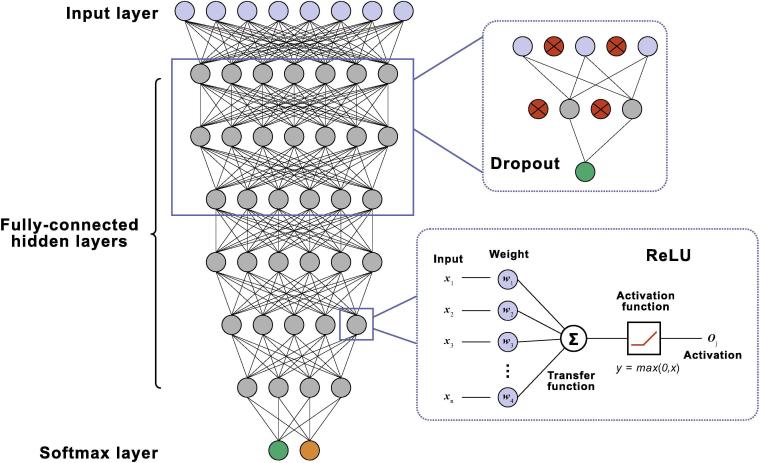
**The deep neural network model of DeepNitro** A total of eight neural levels were implemented. To reduce overfitting, a dropout process was introduced in the first three hidden layers. Additionally, for each dense layer (the fully-connected layer), ReLU was applied to avoid gradient diffusion. Blue circles indicate neurons in the input layer, the green and orange circles are neurons in the output layer. Red circles refer to neurons that are blocked by the dropout approach. ReLU, rectified linear unit.

For the detection of potential nitration or nitrosylation sites, we introduced a deep neural network model in our prediction algorithm. By coarse grid search, we optimized the network as an eight-layer architecture (Figure 1, Figure S1). Specifically, the first layer provided a module for data input, and numeric vectors were directly assigned to the neurons during training and predicting steps. From the second to the seventh layer, each layer was implemented as a fully connected dense layer according to the equation below,

(11) $$o_{j}^{l}=\sigma \left(\sum_{i=1}^{H}w_{i,j}^{l-1}o_{i}^{l-1}+b_{j}^{l-1}\right)$$

where *H* is the number of neurons in the ${\text{(l - 1)}}^{\mathrm{th}}$ layer, ${\text{w}}_{\text{i,j}}^{\text{l - 1}}$ and ${\text{b}}_{\text{i,j}}^{\text{l - 1}}$ are the weight and bias associated with the *j*th neuron, respectively, and $\sigma \left(\cdot \right)$ denotes a non-linear activation function in this neuron. We expected that high-level features might be extracted from the input vector when propagating the signal down these layers. The eighth layer is the output layer.

In probability theory, the output of a softmax function can be used to represent a probability distribution over k different possible outcomes. Therefore, it is widely used in various multiclass classification algorithms, such as softmax regression, naive Bayes classifiers, and artificial neural networks. Specifically, in neural network, softmax function was first introduced by Bridle and his colleagues [42]. Comparing to simple logistic regression, deep neural network with a softmax classifier can vastly improve the performance in the case of not being able to perform feature selection, which is important to find out features that significantly affect the outcome of the classification. In view of this, to achieve a classification of the extracted features, we therefore introduced a softmax classifier in the output layer,

(12) $${\text{o}}_{\text{k}}\;\text{=}\;\frac{{\text{e}}^{{\text{y}}_{\text{k}}}}{\sum_{\text{i = 1}}^{\text{H}}{\text{e}}^{{\text{y}}_{\text{i}}}}$$

where *o_k_* is the output of the *k-*th neuron, representing the observed probability of class *k*; *y_k_* is the associated linear output from the previous hidden layers; and *H* is the total number of output neurons in the softmax layer. Specifically, for all the layers above, the ReLU function (Equation (13)) was adopted as the activation function to avoid gradient diffusion during the training process. The gradient diffusion, also known as vanishing gradient problem, is a challenge in the training of artificial neural network by gradient-based learning methods and backpropagation [43]. In such method, the update of neuron’s weights and biases requires to pass backwards the error signal from the previous layers. When the network is deep enough, the gradient of the loss function would be vanishingly small, effectively preventing the change of the weight. In the worst case, this may completely stop the neural network from further training, which explains the hindered usage of deep neural network in solving complex problems until recently when ReLU [27] function was introduced as a solution. Since the derivative of ReLU is constant at 1 for any input value greater than zero, the error signal would never attenuate when propagating down the network. This makes ReLUs favorable for the complex neural network model, and we can have very deep neural networks with ReLUs without the vanishing gradient problem.

(13) $$\text{f}\left(\text{x}\right)=\left\{\begin{array}{ccc} 0 & \mathrm{for} & \text{x}<0\\ \text{x} & \mathrm{for} & \text{x}⩾0 \end{array}\right)$$

Before using the deep neural network for prediction, we must first optimize the network parameters for the training dataset. To achieve this goal, the negative log-likelihood (Equation (14)) was considered as a loss function in our optimization steps,

(14) $$\text{L}=-\frac{1}{\text{H}}\sum_{i=1}^{H}o_{i}\log\overset{⌢}{\underset{i}{o}}$$

where ${\hat{o}}_{i}$ represents the predicted label, while ${\text{o}}_{\text{i}}$ is the real label. Again, *H* is the total number of output neurons in the final layer. According to Equation (14), a mini-batch gradient descent algorithm is used to update network weights and biases during the back-propagating process. The gradients can then be computed as shown below,

(15) $${\text{w}}^{\text{l}}\leftarrow \text{m}\times {\text{w}}^{\text{l}}-\eta \frac{\partial \text{E}}{\partial {\text{w}}^{\text{l}}}$$

(16) $${\text{b}}^{\text{l}}\leftarrow \text{m}\times {\text{b}}^{\text{l}}-\eta \frac{\partial \text{E}}{\partial {\text{b}}^{\text{l}}}$$

where *η* is the learning rate. The batch size was optimized to be 30 for protein nitration and set at 50 for protein *S*-nitrosylation, respectively. To reduce overfitting, the momentum item was adopted for updating the weights and bias, and both the L1 and L2 regularization were introduced in the loss function (Equation (17)),

(17) $$\text{L = L +}\;\lambda_{\text{1}}\sum \left|\theta \right|\;\text{+}\;\lambda_{\text{2}}\sum \theta^{\text{2}}$$

where *L* is the loss function; *λ_1_* and *λ_2_* are coefficients for *L_1_* and *L_2_* regularization, respectively; and *θ* is the weights and biases in a given layer.

Additionally, the dropout method was also implemented from the second to the fourth layer to improve the generalization capacity for the unknown dataset. Detailed parameter settings are also optimized by coarse grid search and are listed in Table S5.

The aforementioned deep neural network was implemented and trained by the *deeplearning4j* library in JAVA.

### Evaluation of the feature abstraction ability in the deep neural network

To further decipher the underlying mechanism of our constructed models, we have designed the following method to evaluate the feature abstraction abilities in the deep neural network. The abstracted features for each training and test dataset from the second to the seventh layer were computed based on the previously trained model. The abstracted features from each layer were then input into a simple multilayer perceptron (MLP) for training and testing. This simple MLP consisted of four fully-connected layers, and the detailed parameters are listed in Table S6. Using the training dataset, this MLP model was retrained to classify the abstracted features. Next, the performance of the retrained model was evaluated on the independent test dataset, and the AUC value was calculated as criteria for quantifying the abstraction ability of each layer. To compare with other traditional feature selection approaches, the principle component analysis (PCA) was also performed on the raw features. Particularly, in order to avoid any bias, the raw features were compressed to the same dimension as each hidden layer from the deep neural network model. Similarly, the abstraction ability of PCA method was evaluated using the same procedure.

The abstraction ability per unit of feature is computed as below,

(18) $${\text{S}}_{\text{i}}\;\text{=}\;\frac{{\text{A}}_{\text{i}}\;\text{-}\;0.5}{{\text{D}}_{\text{i}}}$$

where *A_i_* is the AUC value of the abstracted features from the *i-*th layer, and *D_i_* is the number of abstracted features at this layer. Theoretically, an AUC value of 0.5 indicates a random classification model, and thus, AUC value greater than 0.5 would contribute to classification. Therefore, we subtract 0.5 from the calculated AUC value to quantify the real contribution, and further divided it by the feature’s dimensionality to compute the abstraction ability per unit of feature.

## Results

### Construction of predictors

We first optimized the length of the flanking regions of a potential nitration or nitrosylation site by traversing the window size *L* from 10 to 50 using a 4-fold cross validation. Then various encoding schemes, including the one-hot binary encoding, PFR, k-space spectrum, and PSSM encoding, were applied to capture the sequence and physiochemical features of the flanking regions. As shown in Figure S2, increases in the window size improved the prediction performance of tyrosine nitration, tryptophan nitration, and *S*-nitrosylation. Taking into account both the prediction accuracy and computational burden, we finally selected a peptide length of 41 aa for subsequent training and prediction. Accordingly, a given nitration or nitrosylation site peptide could be encoded into a 3311-dimensional feature vector, when all the four feature encoding schemes above were used.

**Figure 2:**
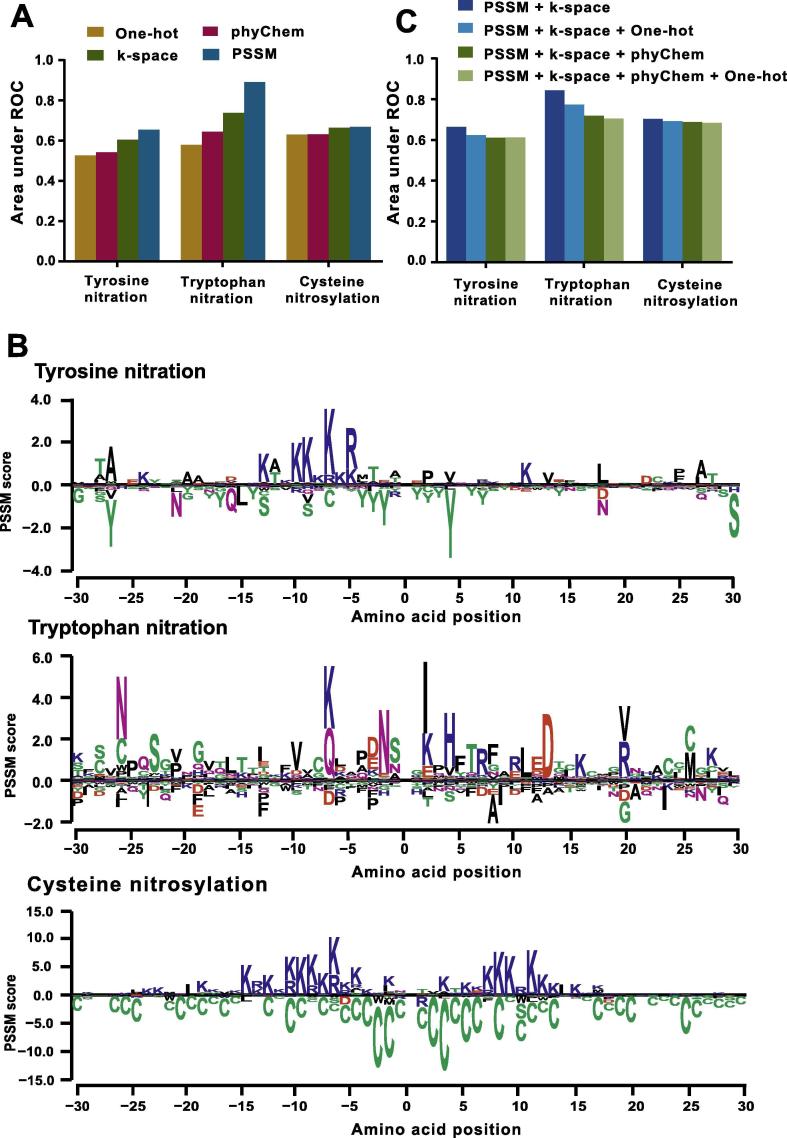
**Establishment of the optimal encoding scheme for DeepNitro** **A.** The prediction capabilities of the four different types of encoding schemes. **B.** The modified PSSM encoding features for protein nitration and nitrosylation. The PSSM scores were calculated according to our modified methods (see Methods section). The amino acid profiles were generated using seq2logo [49] based on the calculated PSSM scores. Position 0 denotes the nitrated or nitrosylated residue. **C.** The evaluation results of different combination of feature-encoding schemes. Feature-encoding schemes were added sequentially according to their prediction capabilities, and 4-fold cross-validation was applied to evaluate their prediction performance. PSSM, position specific scoring matrix.

To capture all available biological properties, we designed a set of feature-encoding schemes for protein nitration and nitrosylation. However, we speculate that different types of features might contribute divergently to the prediction performance, and there might be a dependency relationship present among different features. Thus, we first examined the prediction capacities of the four encoding schemes by 4-fold cross-validation. The evaluation results of tyrosine nitration, tryptophan nitration, and *S*-nitrosylation showed a high coincidence. PSSM and k-space encoding were the two most effective feature capturing schemes for protein nitration and nitrosylation, enabling better classification than the other two schemes (Figure 2A). Although experimental evidence has shown that the formation of protein nitration and nitrosylation is mainly regulated by chemical side reactions produced via NO-related pathways, herein, we unexpectedly observed a weak contribution of physiochemical features to the prediction of potential nitrogen-containing modifications (Figure 2A). Among all the feature-coding schemes, we found that our modified PSSM scheme achieved an outstanding efficacy for extracting underlying features of the protein modification sites that were lack of consensus motifs. Instead of only calculating the conservation degree for positive data, we also took into account the contribution of negative data. In fact, by measuring the subtle differences between positive modification sites and negative residues, we could further extract the underlying rules for classification in comparison to the traditional PSSM method. For example, in our PSSM scheme, we observed that adjacent basic amino acid residues, such as arginine and lysine, were favorable for producing tyrosine nitration, while adjacent aromatic amino acid residues, such as tyrosine, hindered tyrosine nitration (Figure 2B, Figure S2). Similar patterns were also observed for cysteine *S*-nitrosylation. The proximal basic amino acid (arginine or lysine) residues showed a striking preference for positive data. Additionally, the cysteine residues located around the flanking region were deleterious to the modification (Figure 2B, Figure S2). Interestingly, the amino acid preference for tryptophan nitration seemed to be more site-specific compared with the other two types of modifications. The aliphatic or basic amino acid residues at positions −7, −2, +2, +4, and +13 contributed positively to the modification of the nitro-group on tryptophan. In contrast, the alanine residue at position +8 and isoleucine residue at position +9 contributed negatively to tryptophan nitration (Figure 2B, Figure S2).

Based on the observations above, we next sought to test the prediction performance for different combinations of the feature-encoding schemes. We evaluated the prediction capacities of the combined schemes using 4-fold cross-validation by adding the schemes sequentially into the prediction model based on the order of their contributions. As expected, using a combination of schemes could improve the prediction accuracy for tyrosine nitration and cysteine nitrosylation (Figure 2C). In particular, incorporation of the PSSM and k-space schemes exhibited optimal performance. Therefore, herein we chose PSSM and k-space as the final feature-encoding schemes and constructed a 2040-dimensional numeric vector for both tyrosine nitration and cysteine nitrosylation prediction. For the tryptophan nitration, there seems to be a different trend between the scheme combinations and the prediction performance. PSSM encoding showed the strongest capacity for prediction, whereas the integration of other features seemed to weaken its prediction capacity. Notably, the prediction performance of the constructed model decreased with increased number of integrated schemes (Figure 2C). Due to the limited number of known nitration sites available in our training dataset, the application of multiple features during the encoding process will introduce extra noise for classification, which may hinder the improvement of the prediction accuracy. Therefore, to obtain the most appropriate prediction model, we selected only the PSSM as the final scheme for feature encoding of tryptophan nitration. A 40-dimension feature vector was inputted to the deep neural network model for training and prediction.

Based on the aforementioned feature selection strategies, we introduced a deep neural network model to construct the predictor called DeepNitro for the detection of potential nitration or nitrosylation sites (Figure 1).

### Evaluation of the feature abstraction abilities in the constructed predictors

In deep learning-based method, as a benefit of its hierarchical architecture, input features can be precisely abstracted along the successive levels and thereby discovering informative patterns for subsequent classification or regression tasks. The following method was developed to evaluate the feature abstraction abilities of our constructed predictors. Firstly, we extracted the output signals from each hidden layer as the abstracted features, and input them into a simple multilayer perceptron (MLP) to test their classification performance. Using our collected dataset, we retrained the MLP model and calculated the AUC value under the independent testset. To compare with other feature selection approaches, the principle component analysis (PCA) was also performed on the raw input data and the selected components were then propagated forward the same MLP to compute its performance. Expectedly, the abstracted features selected from the nitration and nitrosylation models all outperformed those from the PCA method, suggesting that our method have a better feature abstraction abilities than the traditional approaches (Figure S3). We further computed the abstraction efficiency per unit of feature for both the deep neural network and PCA method. Obviously, the abstraction efficiencies in each hidden layer showed that deep neural network is able to extract effective features by transforming signals through successive layers. As the number of layers increased, more informative features were obtained. Moreover, compared to PCA method, deep neural network can preserve more information under the same compression ratio.

Taken together with the observations above, the application of a deep neural network in our study could automatically extract high-level recognition patterns for protein post-translational modifications, and help to eliminate irrelevant features or reduce noise in the training process.

### Evaluation of the prediction performance

**Figure 3:**
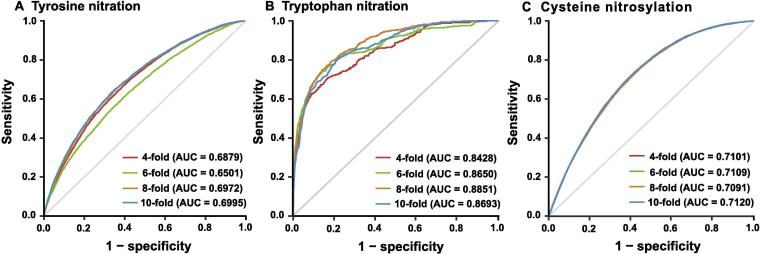
**Performance evaluations of the prediction models for tyrosine nitration, tryptophan nitration, and the *S*-nitrosylation** The 4, 6, 8, and 10-fold cross-validations were performed on the tyrosine nitration (**A**), the tryptophan nitration (**B**), and the cysteine nitrosylation (**C**) models.

To evaluate the prediction performance of DeepNitro, we performed 4-, 6-, 8-, and 10-fold cross-validation of the training dataset. As a result, DeepNitro showed an acceptable performance in n-fold cross-validation with the area under the ROC curve (AUC) close to 0.7 for tyrosine nitration (Figure 3A). For tryptophan nitration, the AUC values were mostly greater than 0.85, indicating a satisfactory prediction performance (Figure 3B). For *S*-nitrosylation, all the tested AUC values were greater than 0.7 (Figure 3C). Furthermore, for both nitration and nitrosylation, the ROC curves of the 4-, 6-, 8-, and 10-fold cross-validations were very close to each other, supporting the robustness of our constructed predictors. Since the positive and negative datasets were highly imbalanced in our training dataset, we then calculated the precision-recall curves to further evaluate the performance of our prediction models (Figure S4). The results further indicate that DeepNitro is accurate and robust in predicting novel nitration and nitrosylation sites, even in the case of an imbalanced data dataset.

In our prediction models, we expect that application of deep neural network may help to uncover the underlying sequence features of protein nitration and nitrosylation from the training dataset. To prove this point, we further compared our deep neural network models to other shallow machine learning methods for their abilities to interpret the training dataset. The two most widely-adopted algorithms, support vector machine (SVM) and random forest (RF), were compared and a 4-fold cross-validation was carried out to evaluate their performance. As shown in Figure S5, applying the eight-layer deep neural network substantially improved the prediction capability for tyrosine nitration and *S*-nitrosylation over SVM and RF, indicating that the deep neural network models are more powerful in interpreting the underlying information for training dataset. However, for tryptophan nitration, random forest seemed to be the optimal algorithm. Generally speaking, training deep neural network typically requires a certain amount of training dataset to maintain the robustness and accuracy of the model. But in tryptophan nitration, the sample size is quite small, and therefore, limiting the performance of deep neural network. The advantage of deep neural network can be further demonstrated as the training dataset expands in the near future.

**Figure 4:**
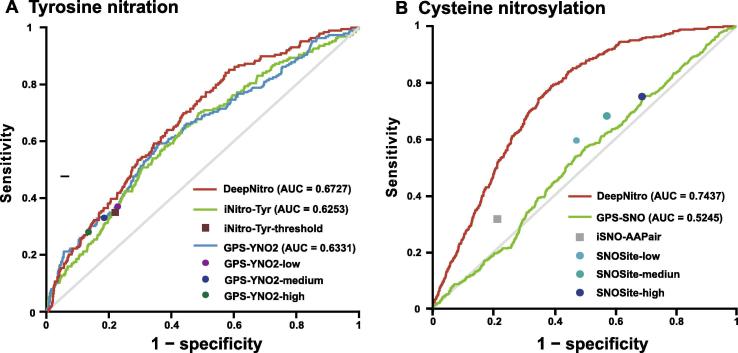
**Performance comparison of the tyrosine nitration and the *S*-nitrosylation prediction models for the independent benchmarking dataset** An independent test set was used to evaluate the prediction capability of DeepNitro and other existing tools on protein nitration (**A**) and nitrosylation sites (**B**). The default prediction thresholds of each compared tool were also marked on the ROC curves. Since three prediction thresholds with low, medium and high stringencies were provided in GPS-YNO2 and SNOSite, we separately marked them on the corresponding ROC curves. ROC, receiver operating characteristic curve.

To rigorously evaluate the prediction performance of DeepNitro, we next compared it with other state-of-art predictors using an independent dataset. For tyrosine nitration, we selected iNitro and our previous tool, GPS-YNO2, for comparison. To ensure that the comparison was unbiased, all the different thresholds for these tools were used. The evaluation results of DeepNitro in the independent dataset agreed well with those from the n-fold cross-validation, indicating that our model provides robust results for new data. In comparison to other available tools, DeepNitro also showed superior prediction performance (Figure 4A). For *S*-nitrosylation, iSNO-AAPair, SNOsite, and GPS-SNO were selected to perform the comparison. Since iSNO-AAPair and SNOsite did not provide prediction scores for all potential cysteine residues, we could only compute the prediction performance under their default thresholds. As presented in Figure 4B, our model achieved the highest AUC value of 0.7437, outperforming other prediction software. To our knowledge, our study is the first attempt to establish a prediction model for tryptophan nitration for the biology community, and therefore a performance comparison was not performed for tryptophan nitration.

### Development of the DeepNitro web server

**Figure 5:**
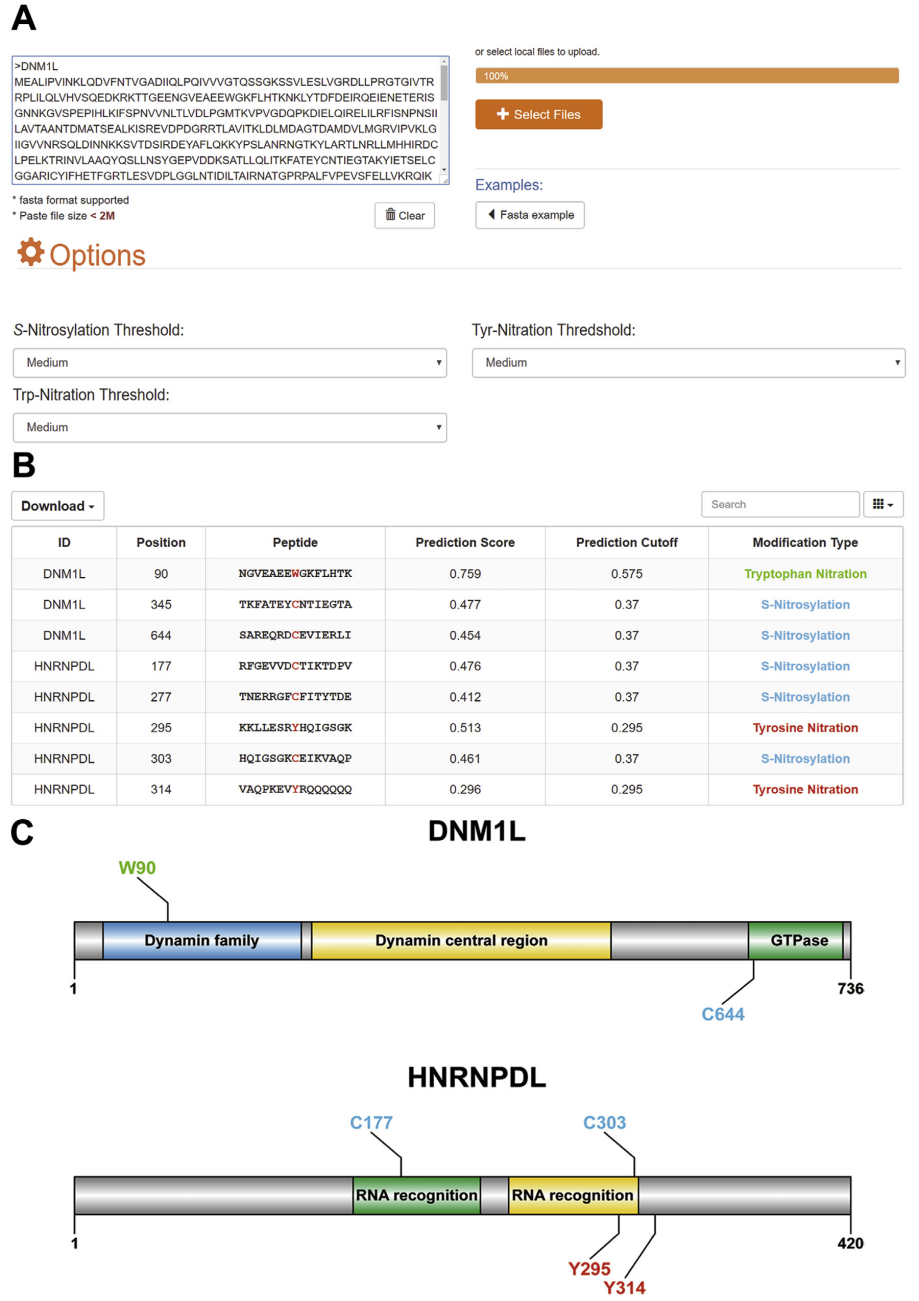
**A snapshot of the DeepNitro server** **A.** The main interface of DeepNitro. Protein sequences can be input into the text area or uploaded as a single FASTA file. Thresholds with high, medium, and low stringencies are provided in the options panel. Detailed information for the predicted modification sites, such as protein name, modified position, flanking peptide, prediction score, and prediction threshold, is listed in the interactive table (**B**) and visualized using IBS and InterProScan (**C**). DNM1L, dynamin-1-like protein; HNRNPDL, heterogeneous nuclear ribonucleoprotein D-like; IBS, illustrator for biological sequences.

To facilitate the use of our prediction models, we next developed an online predictor called DeepNitro for the community, which is freely available at http://deepnitro.renlab.org. DeepNitro only requires protein sequences to run a prediction. The prediction of tyrosine nitration, tryptophan nitration, and cysteine nitrosylation are well supported in our predictor, and users can select the modification types of their interest in the options panel. To balance the prediction accuracy of each modification type, we selected three thresholds with high, medium, and low stringencies based on the evaluation results (Figure 5A). The detailed performance values under these three thresholds are shown in Table S7.

After the query sequences are submitted to DeepNitro, users can check its running status in the result panel in real time. When the prediction is complete, a button that links out to the result page automatically appears. Figure 5B provides a snapshot for the result page of human dynamin-1-like (DNM1L) protein and heterogeneous nuclear ribonucleoprotein D-like (HNRNPDL) protein. The prediction position, score, and modification type of the input proteins were first listed in an interactive table, which allows the users to easily search and sort the results. Remarkably, to facilitate a further analysis of the protein function, we also implemented an automatic pipeline for visualizing the prediction results. By integrating IBS [44] and InterProScan [45] into the web server, DeepNitro can present the graphical representation of the input proteins together with their predicted sites and domain organization in the visualization panel (Figure 5C). In order to allow a mass prediction of protein nitration and nitrosylation sites, a standalone package, which is available at http://deepnitro.renlab.org, was also developed. Like the web server, the prediction of multiple modification types and visualization of the predicted results was supported.

Besides the functionalities above, we also integrated a database covering all the nitration and nitrosylation sites we collected into the web server. By searching with annotation keywords or protein sequences, users can easily get access to the modification data of interest and perform further functional analysis to uncover the potential role of protein nitration and nitrosylation.

## Discussion

Protein nitration and nitrosylation are widespread modifications that modulate diverse aspects of cellular functions. Unlike other PTMs, nitration and nitrosylation are induced by a series of chemical reactions rather than enzymatic processes. As a consequence, this kind of modification usually lacks consensus motifs, making it difficult to predict the exact sites using bioinformatics algorithms. To overcome this challenge, we present a novel computational tool, DeepNitro, for predicting potential nitration and nitrosylation sites. First, we constructed new schemes for encoding the potential modification sites based on primary sequence features. Then, the deep learning algorithm was applied for model training and prediction.

DeepNitro shows superior performance for both protein nitration and nitrosylation compared with existing tools. Its prediction capability is enhanced mainly by the new encoding schemes adopted in this study, especially the PSSM method. In contrast to the traditional process, our method not only measures the conservation in positive data, but also takes into account the comparison of residue preference between positive and negative data. The subtle differences between positive and negative data may act as a key factor to improve the distinguishing capability of our model.

In addition to the encoding schemes, a new model training method is also introduced in our study. Recently, the deep artificial neural network has received increasing attention in the field of machine learning. By propagating raw data down the deep networks, underlying features and highly complicated functions can be effectively encapsulated, increasing the classification and regression capabilities for the input data. Currently, the deep neural network has been shown to improve performance in image [46] and speech recognition [32], natural language understanding [47], and most recently, in computational biology [34], [35]. As we expected, the application of the deep neural network in the current study has introduced remarkable performance gains into our model. The deep neural network allows us to better handle high-dimensional encoding vectors by training complex networks with multiple layers that capture their internal relationship. Compared to other traditional machine learning algorithms, application of this new method can discover high-level features and increase interpretability of protein nitration and nitrosylation.

Although promising performance was obtained using DeepNitro, there is still room for refinement. First, to reduce the computational burden, we only considered the primary sequence feature in the current algorithm. Recent studies have indicated that the tertiary structure is another key feature for determining the occurrence of protein nitration or nitrosylation [38]. Therefore, considering sequence features only will introduce bias into the prediction model. Consequently, we will further introduce a structural encoding scheme, such as features for peptide secondary and three-dimensional structures, in our future versions. Second, novel deep learning architectures will also be applied in the next version of DeepNitro to improve its performance. We are currently working to predict the potential nitration and nitrosylation sites through a deep fully-connected network, which neglects the contextual dependencies of a given residue [48]. To measure the contextual dependencies in nitration and nitrosylation site prediction, the recurrent neural network (RNN) will be integrated in future developments. With the help of RNN model in measuring sequence contextual dependency, we expect that such an advanced architecture can greatly improve the prediction capability of non-consensus protein modifications such as nitration and nitrosylation.

## Authors’ contributions

JR and ZZ conceived the project and supervised the study. YBX designed the algorithm and drafted the manuscript. XL and YL implemented the algorithm and performed the evaluation. LC developed the software and established the web service. WM, JH, JC, YZ, and YX performed the data analysis and help to draft the discussion section. All authors read and approved the final manuscript.

## Competing interests

The authors have declared no competing interests.

## Supplementary material

Supplementary Figure S1 **The optimal flanking regions for each feature-encoding scheme** Shown are the selected flanking regions for tyrosine nitration (**A**), tryptophan nitration (**B**), and S-nitrosylation sites prediction (**C**) using different feature-encoding schemes. PSSM, position specific scoring matrix; ROC, receiver operating characteristic curve.

Supplementary Figure S2 **The calculated amino acid preferences** Amino acid preferences were calculated using a modified PSSM approach and presented in the heat maps for tyrosine nitration (**A**), tryptophan nitration (**B**), and cysteine nitrosylation (**C**). The calculated PSSM scores are presented in a color gradient from blue to red for values ranging from low to high.

Supplementary Figure S3 **The abstraction abilities of the tyrosine nitration and cysteine nitrosylation prediction models calculated from the independent test set** The abstraction abilities quantified using AUC values were evaluated for tyrosine nitration (**A**) and cysteine nitrosylation (**B**). Also, the abstraction abilities per unit of feature were also calculated for tyrosine nitration (**C**) and cysteine nitrosylation (**D**).

Supplementary Figure S4 **The precision-recall curves of the DeepNitro models** The precision-recall curves of tyrosine nitration (**A**), tryptophan nitration (**B**), and cysteine nitrosylation (**C**) by 4, 6, 8, 10-fold cross-validation.

Supplementary Figure S5 **The performance comparison between deep neural network and traditional shallow machine learning algorithm** The prediction performance for tyrosine nitration (**A**), tryptophan nitration (**B**), and cysteine nitrosylation (**C**) using different algorithms was compared by 4-fold cross-validation. SVM, support vector machine.
