# Supplementary figures and images for: DeepNitro: Prediction of Protein Nitration and Nitrosylation Sites by Deep Learning
Source: Genomics Proteomics Bioinformatics. 2018 Sep 27;16(4):294–306. doi: 10.1016/j.gpb.2018.04.007 (PMC6205083; doi:10.1016/j.gpb.2018.04.007)

**A Tyrosine nitration**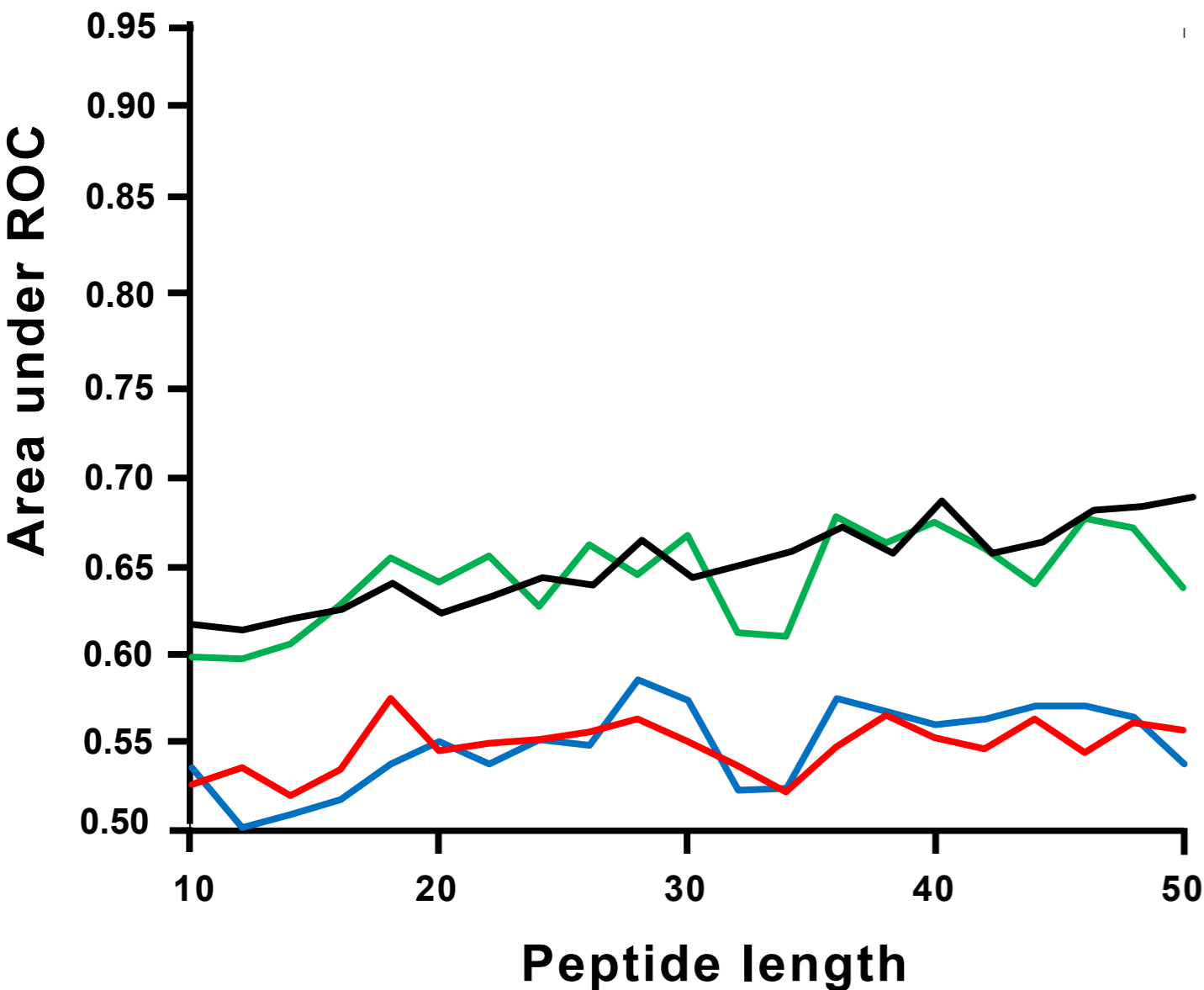**B Tryptophan nitration**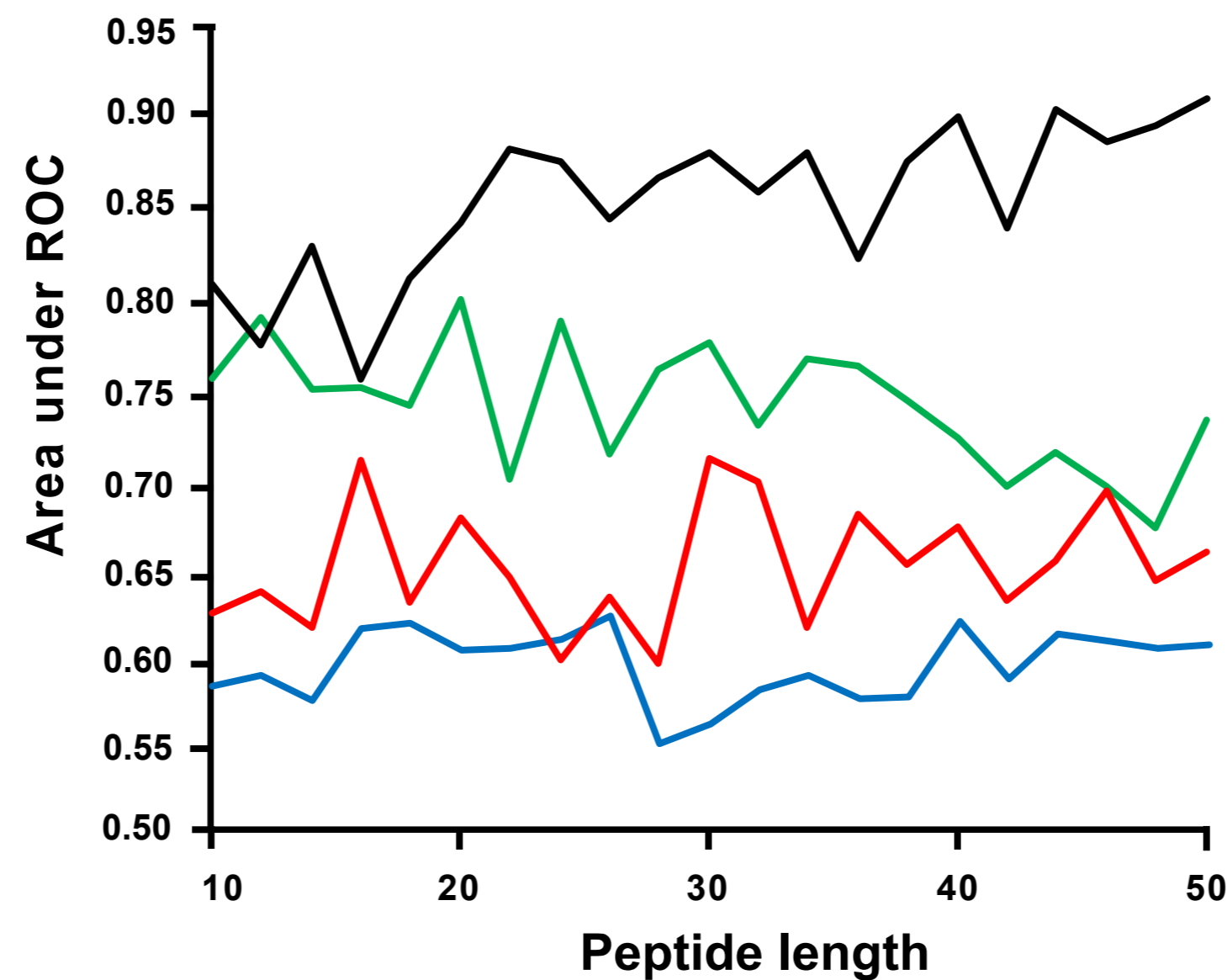**C Cysteine nitrosylation**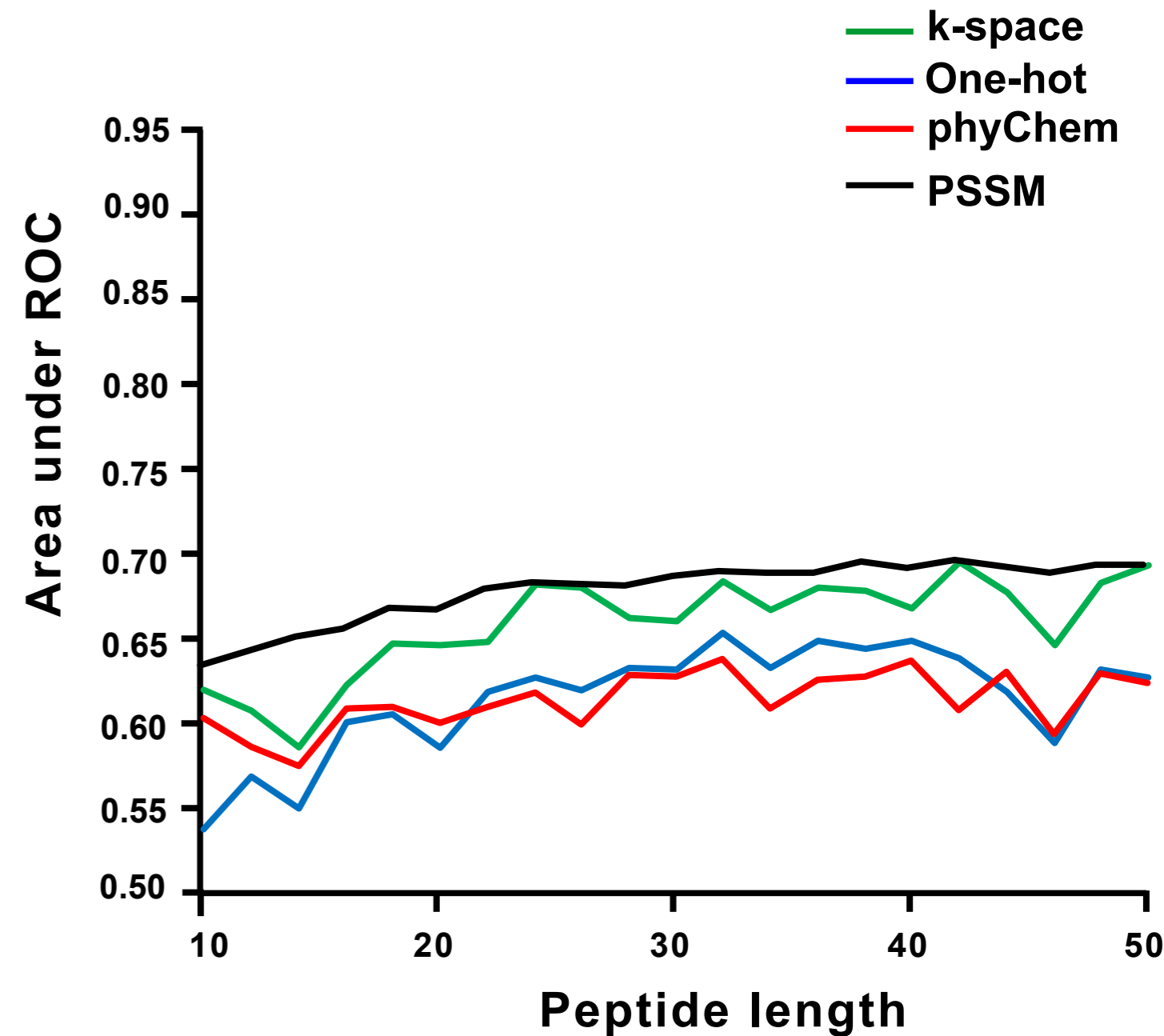

Supplement: Supplementary Figure S1 — The optimal flanking regions for each feature-encoding scheme Shown are the selected flanking regions for tyrosine nitration (A), tryptophan nitration (B), and S-nitrosylation sites prediction (C) using different feature-encoding schemes. PSSM, position specific scoring matrix; ROC, receiver operating characteristic curve. [file mmc1.pdf]

## A Tyrosine nitration

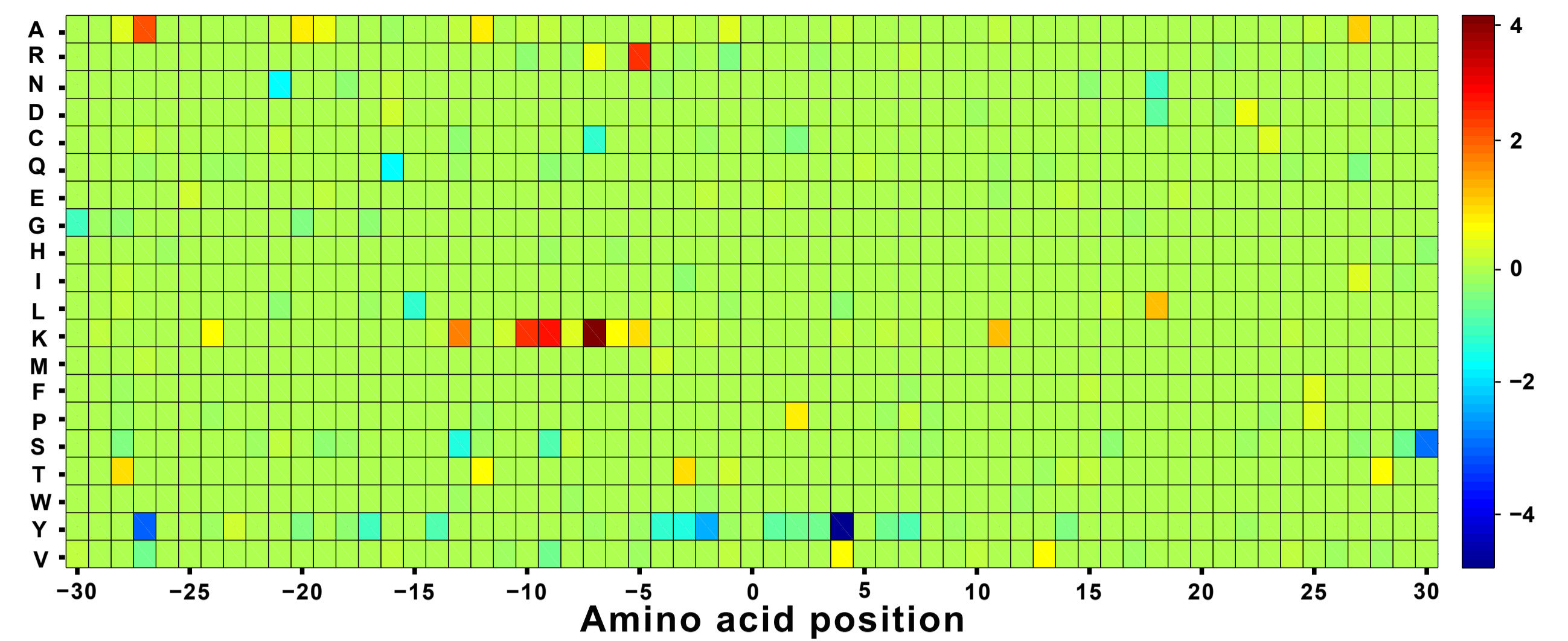

## B Tryptophan nitration

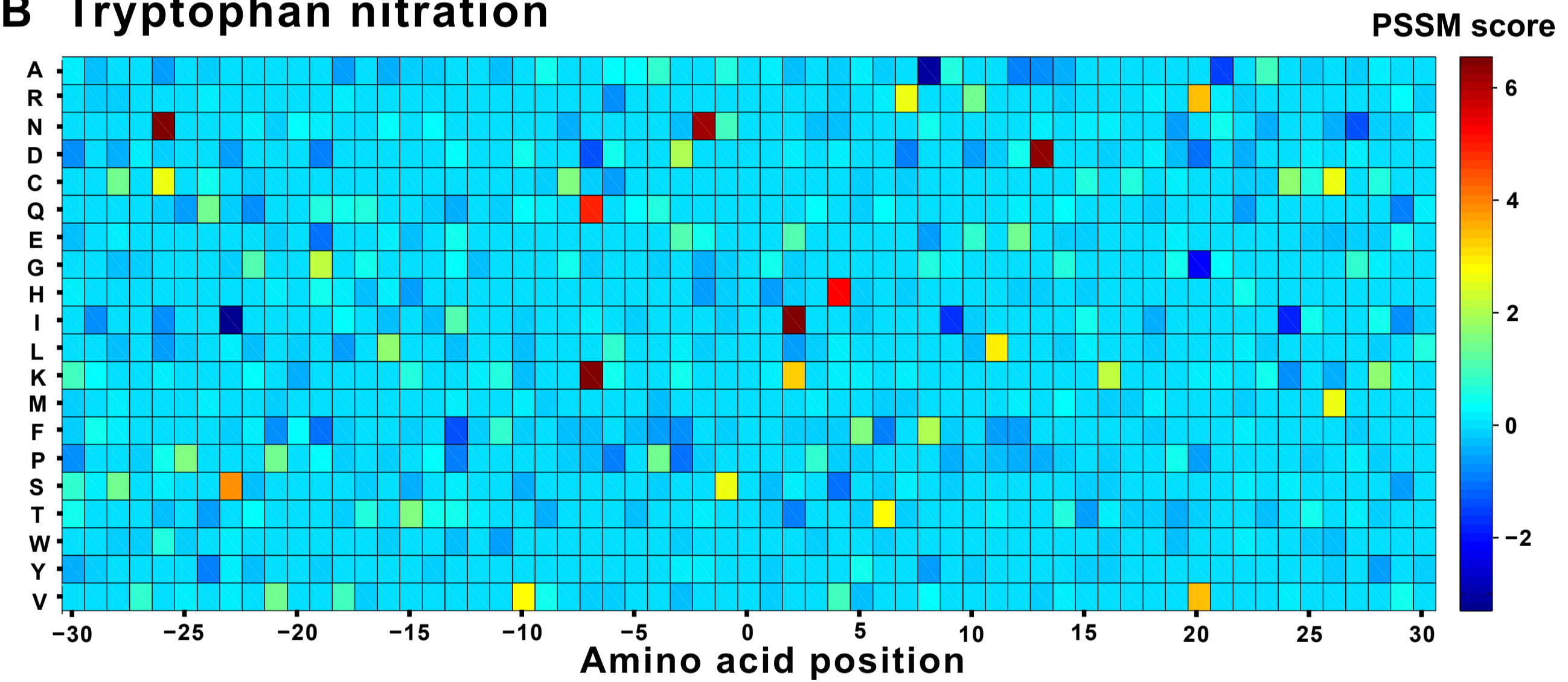

## C Cysteine nitrosylation

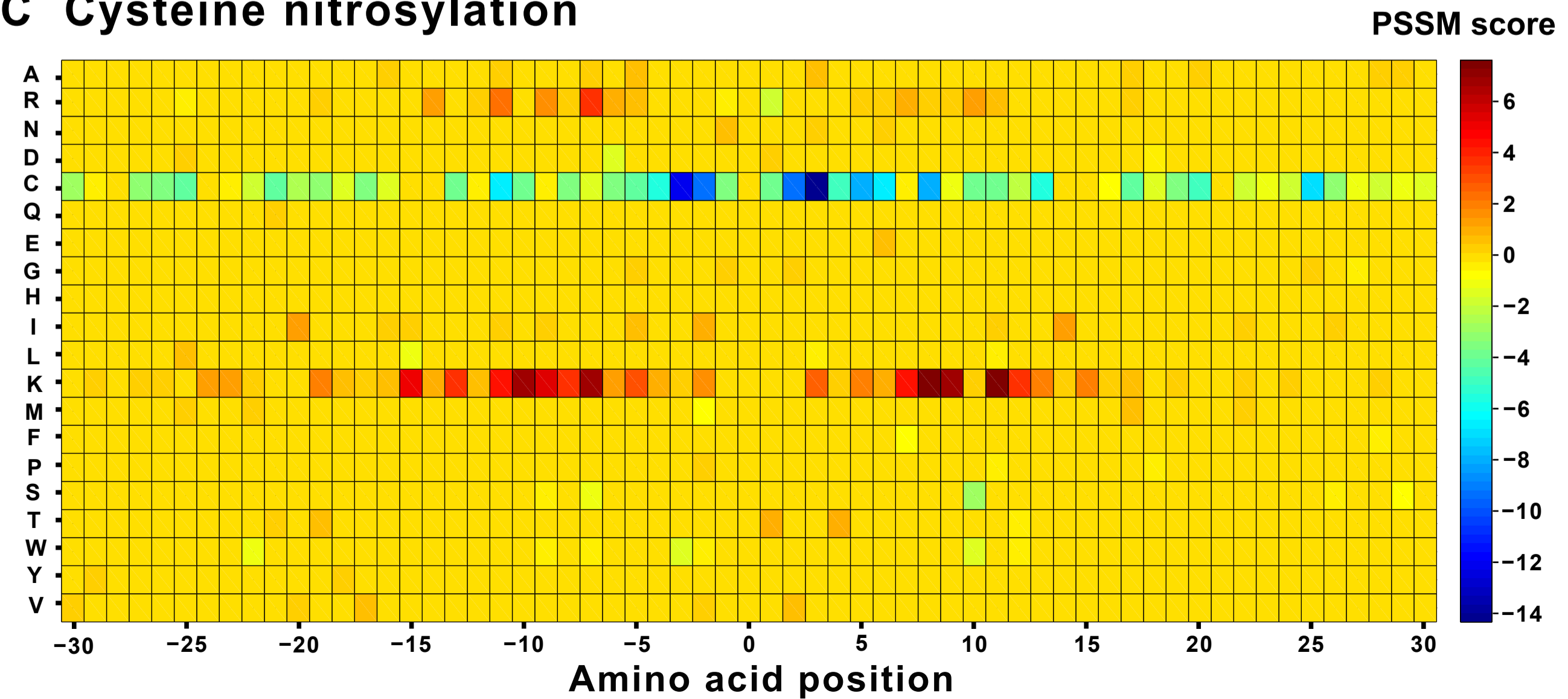

Supplement: Supplementary Figure S2 — The calculated amino acid preferences Amino acid preferences were calculated using a modified PSSM approach and presented in the heat maps for tyrosine nitration (A), tryptophan nitration (B), and cysteine nitrosylation (C). The calculated PSSM scores are presented in a color gradient from blue to red for values ranging from low to high. [file mmc2.pdf]

**A Tyrosine nitration**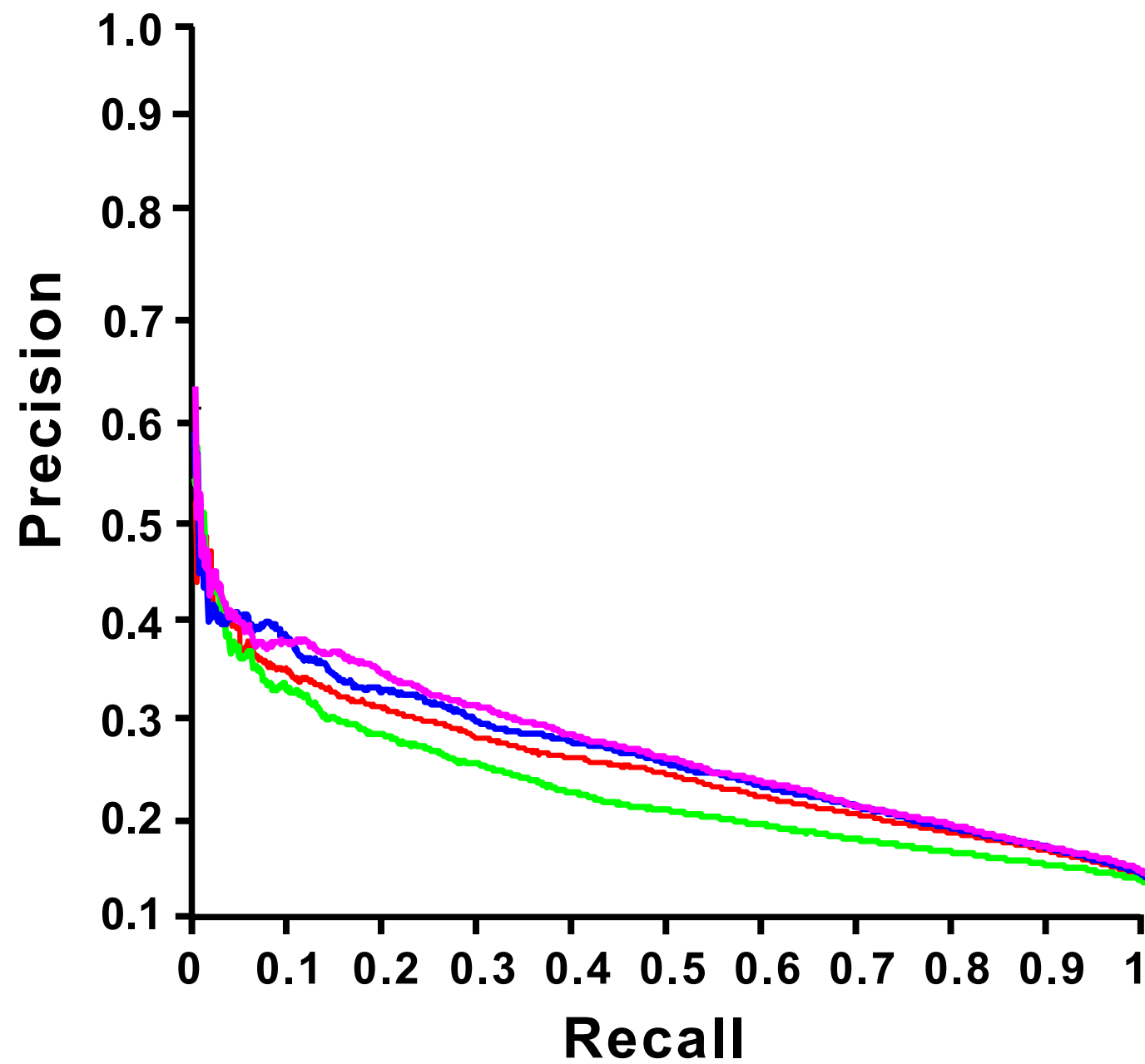**B Tryptophan nitration**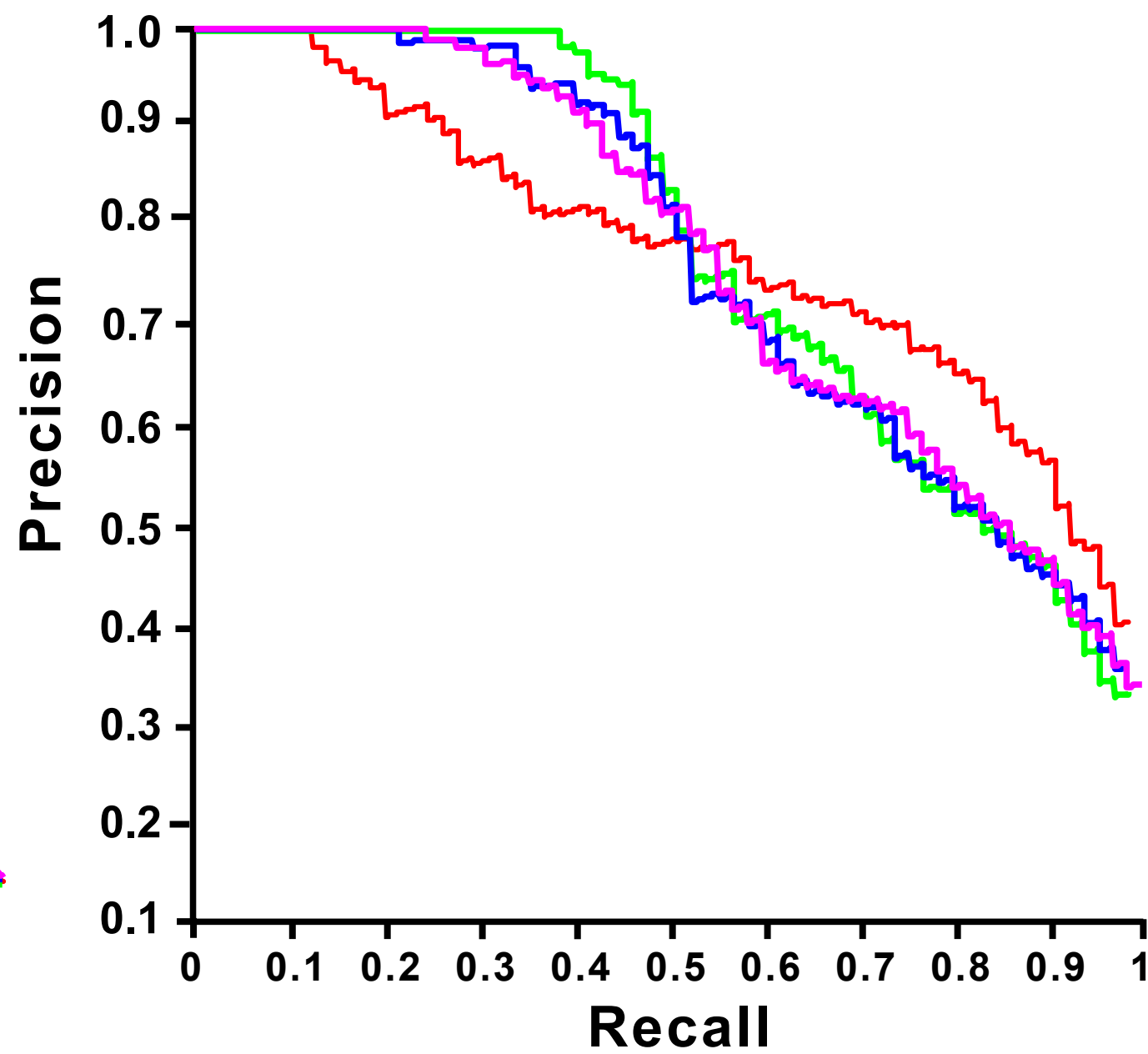**C Cysteine nitrosylation**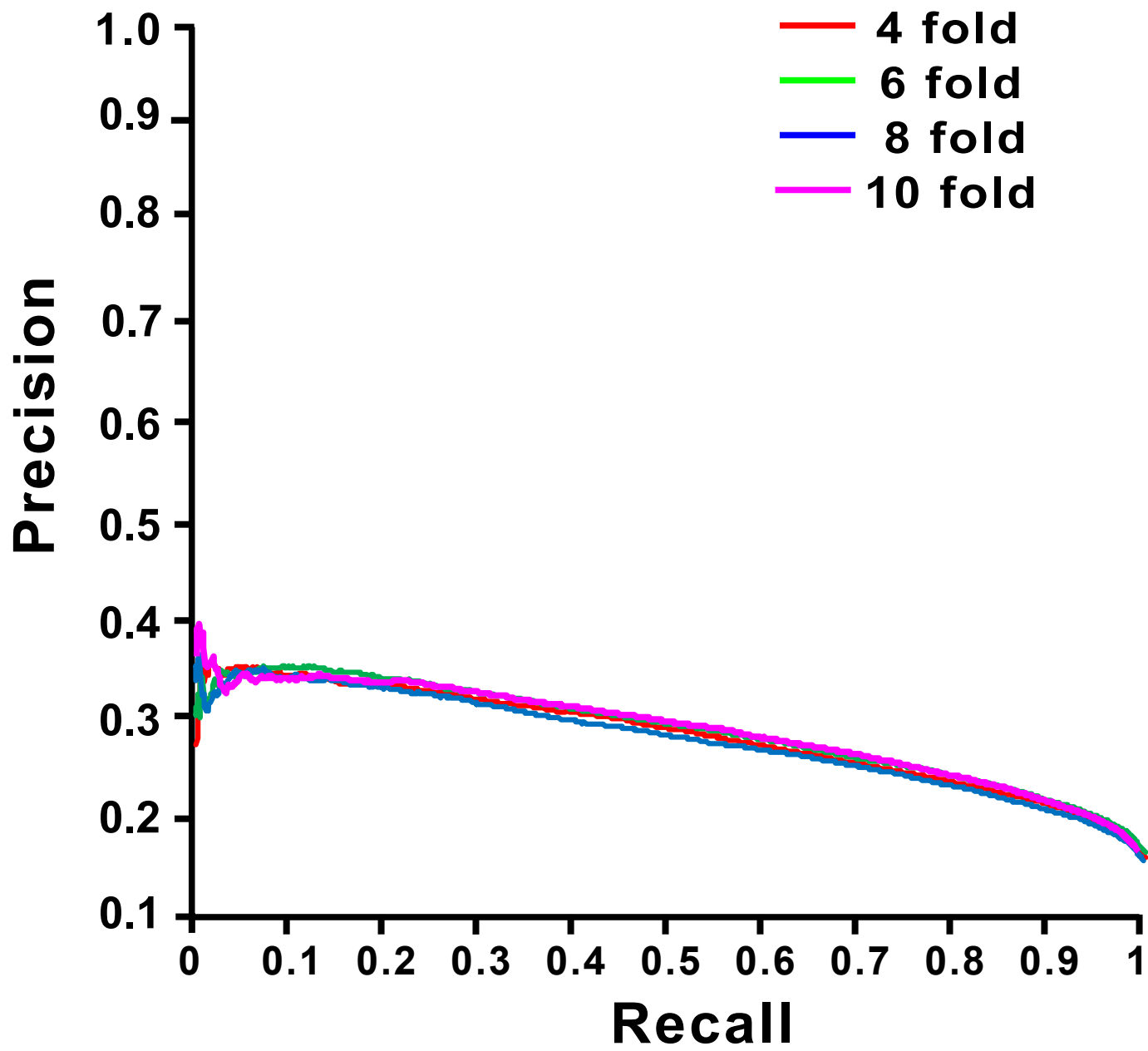

Supplement: Supplementary Figure S4 — The precision-recall curves of the DeepNitro models The precision-recall curves of tyrosine nitration (A), tryptophan nitration (B), and cysteine nitrosylation (C) by 4, 6, 8, 10-fold cross-validation. [file mmc4.pdf]

**A Tyrosine nitration**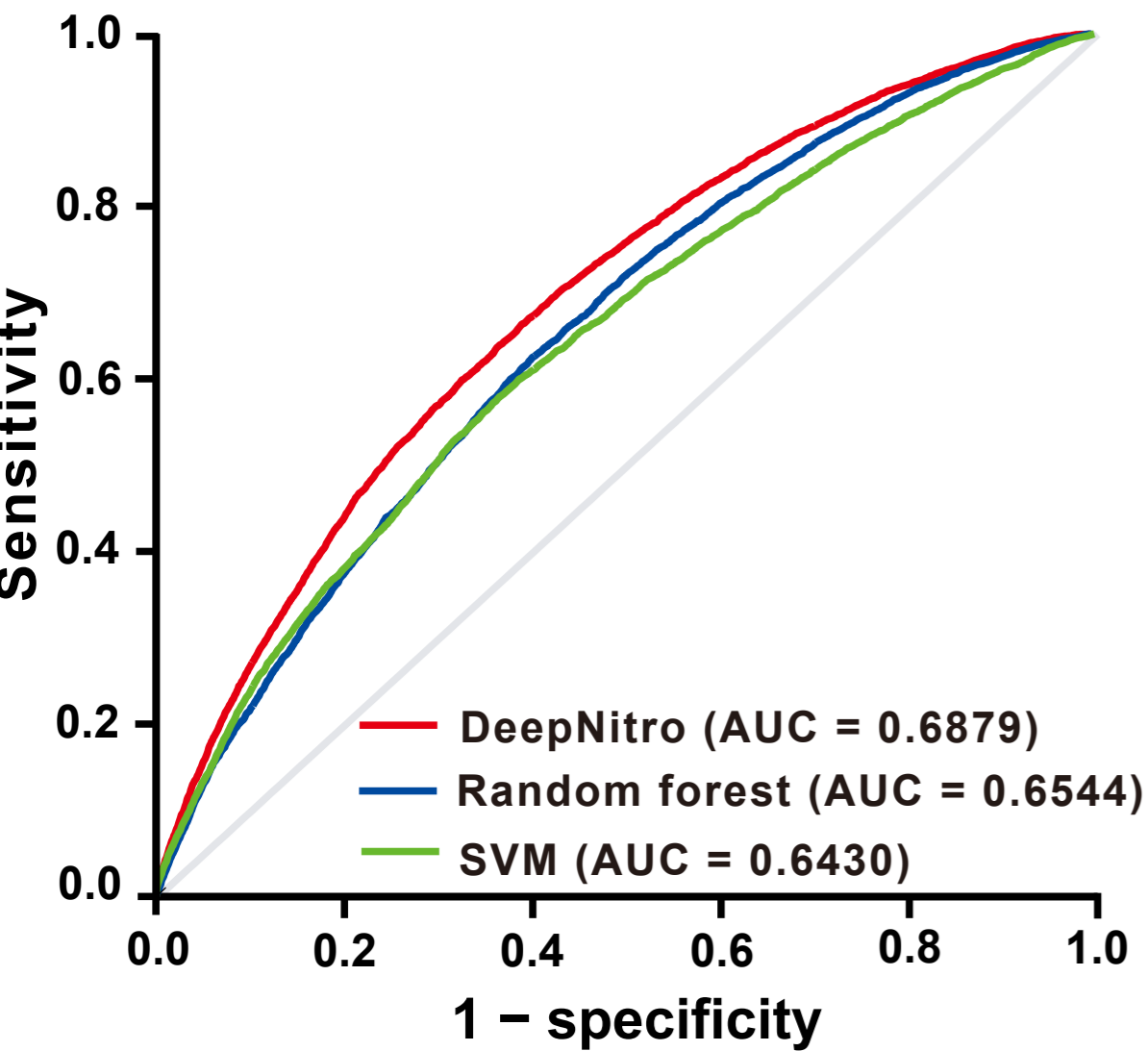**B Tryptophan nitration**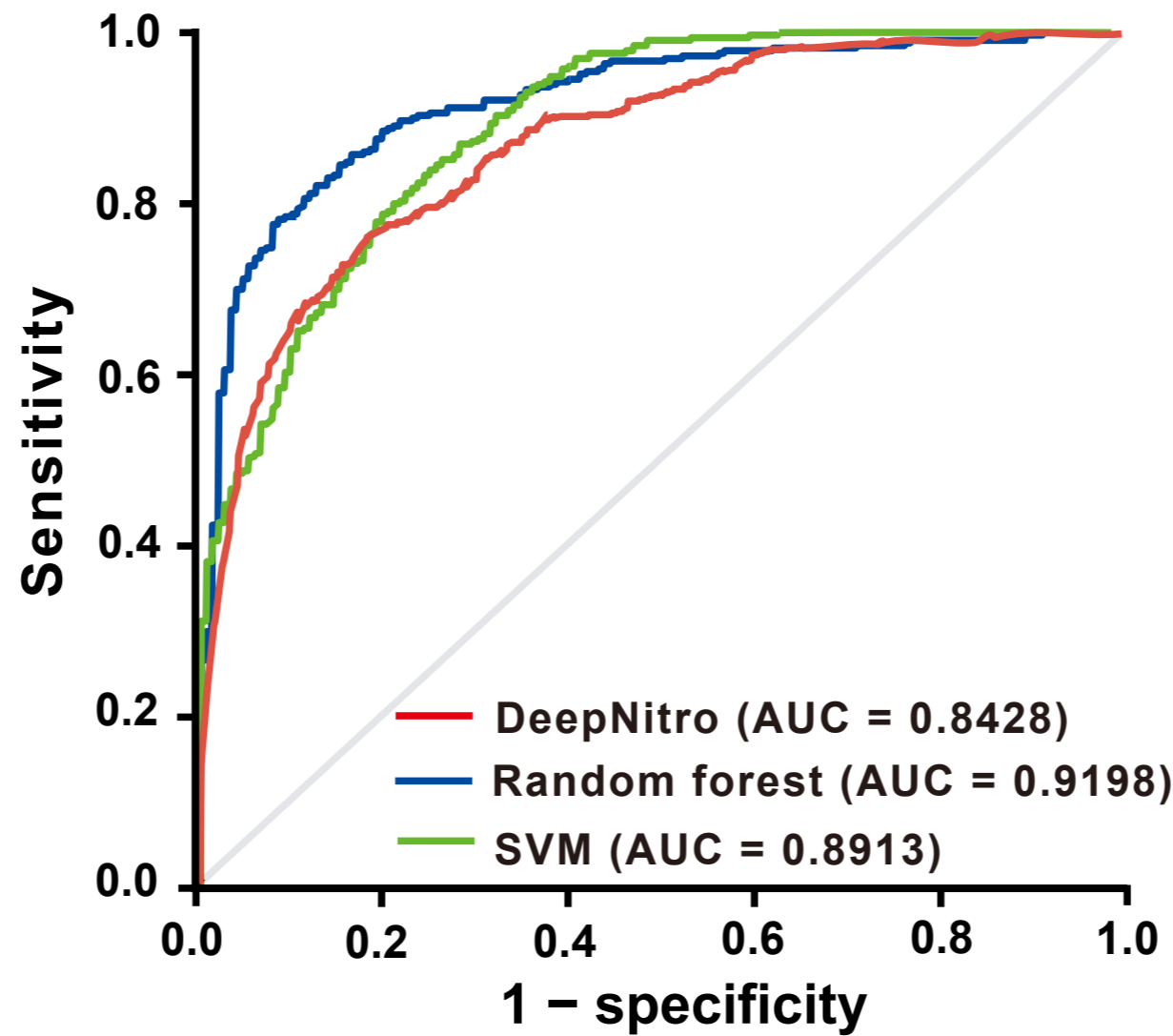**C Cysteine nitrosylation**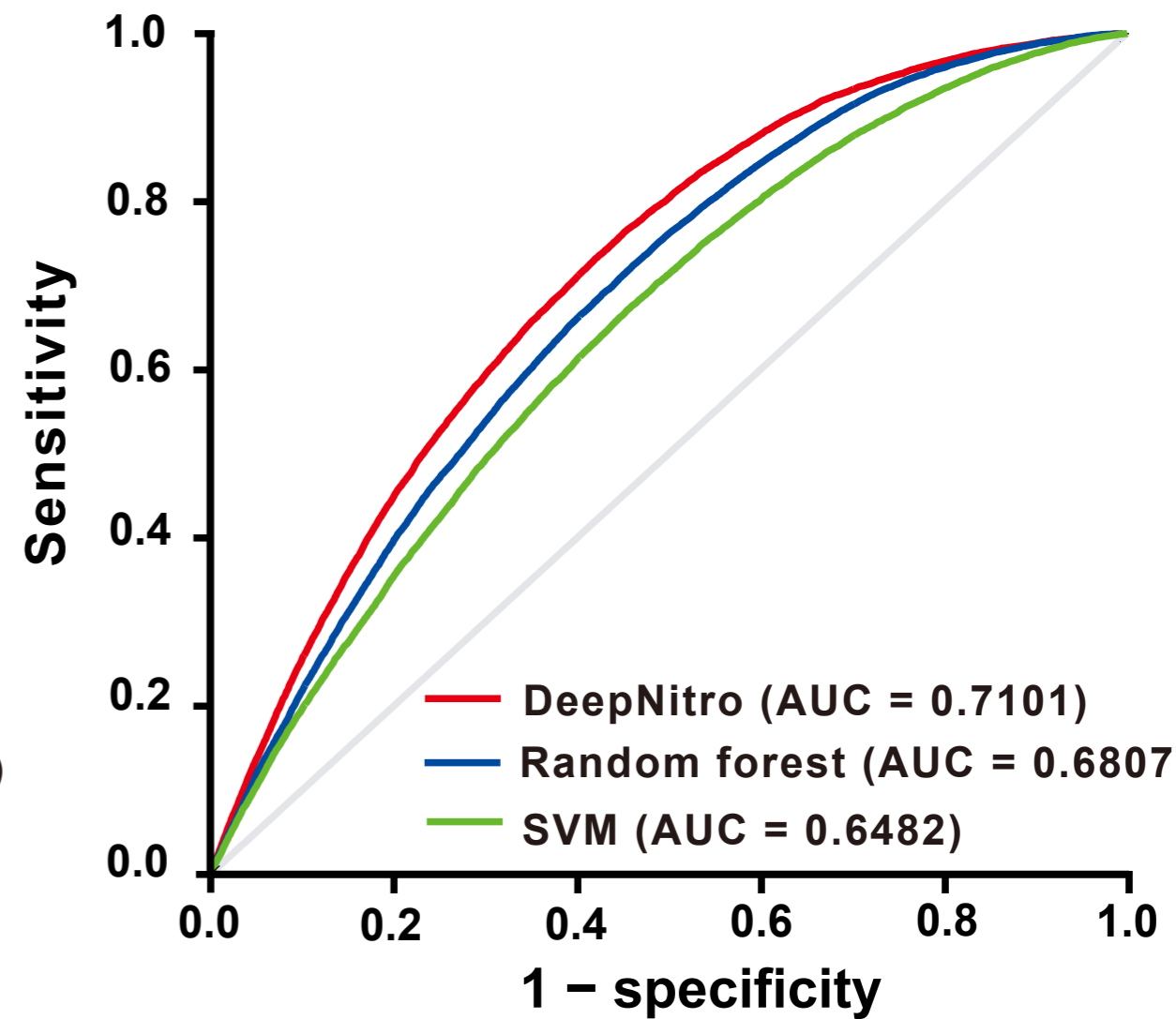

Supplement: Supplementary Figure S5 — The performance comparison between deep neural network and traditional shallow machine learning algorithm The prediction performance for tyrosine nitration (A), tryptophan nitration (B), and cysteine nitrosylation (C) using different algorithms was compared by 4-fold cross-validation. SVM, support vector machine. [file mmc5.pdf]
