# Supplementary material for: DeepNitro: Prediction of Protein Nitration and Nitrosylation Sites by Deep Learning
Source: Genomics Proteomics Bioinformatics. 2018 Sep 27;16(4):294–306. doi: 10.1016/j.gpb.2018.04.007 (PMC6205083; doi:10.1016/j.gpb.2018.04.007)
Supplement: Supplementary Figure S3 — The abstraction abilities of the tyrosine nitration and cysteine nitrosylation prediction models calculated from the independent test set The abstraction abilities quantified using AUC values were evaluated for tyrosine nitration (A) and cysteine nitrosylation (B). Also, the abstraction abilities per unit of feature were also calculated for tyrosine nitration (C) and cysteine nitrosylation (D). [file mmc3.pdf]

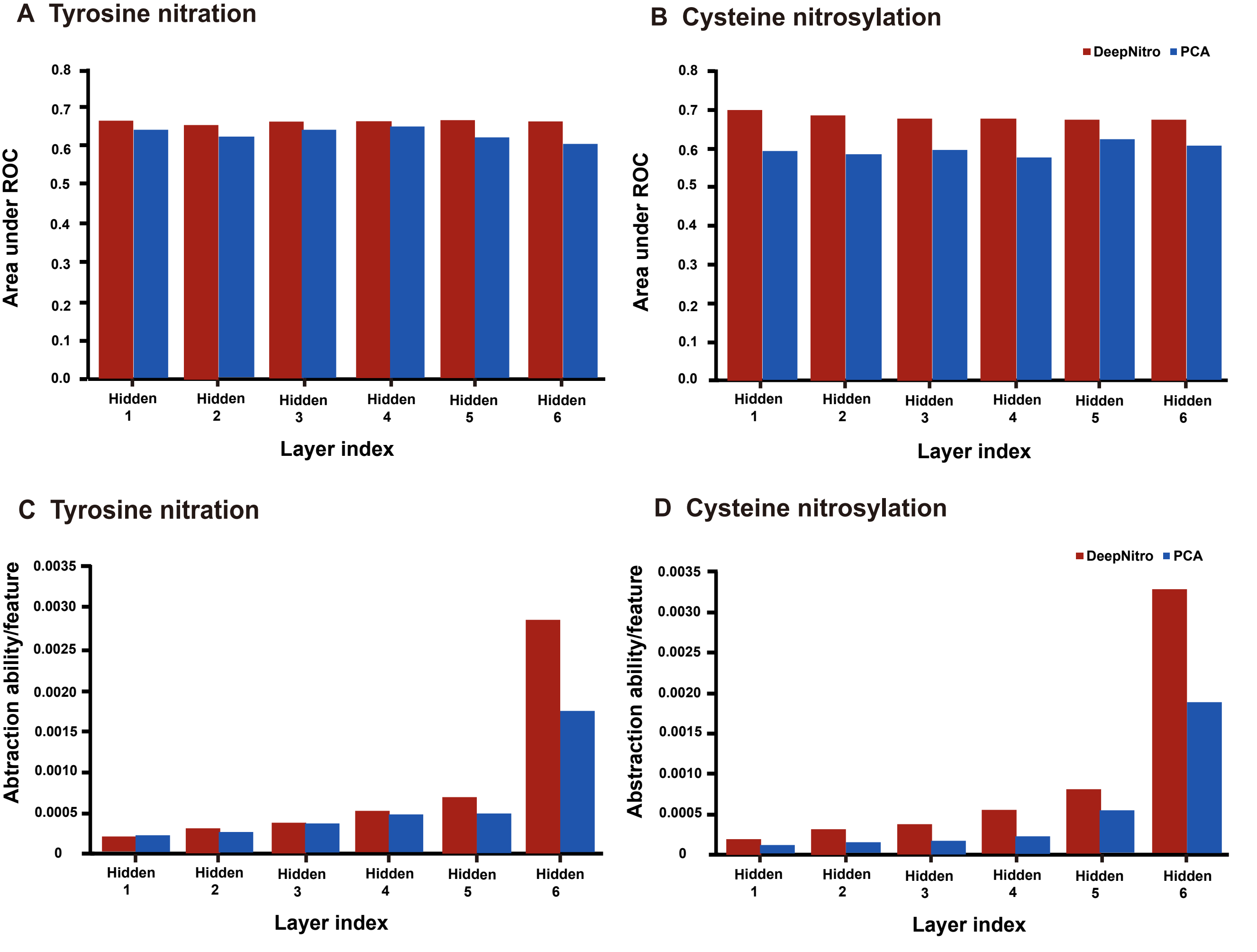

A Tyrosine nitration
B Cysteine nitrosylation
C Tyrosine nitration
D Cysteine nitrosylation
DeepNitro
PCA
Area under ROC
Abtraction ability/feature
Abstraction ability/feature
0.0
0.1
0.2
0.3
0.4
0.5
0.6
0.7
0.8
0
0.0005
0.0010
0.0015
0.0020
0.0025
0.0030
0.0035
Hidden 1
Hidden 2
Hidden 3
Hidden 4
Hidden 5
Hidden 6
Layer index
