# Supplementary material for: DeepNitro: Prediction of Protein Nitration and Nitrosylation Sites by Deep Learning
Source: Genomics Proteomics Bioinformatics. 2018 Sep 27;16(4):294–306. doi: 10.1016/j.gpb.2018.04.007 (PMC6205083; doi:10.1016/j.gpb.2018.04.007)
Supplement: Supplementary Table S3 [file mmc8.docx]

**Table S3 PFR used to encode the physicochemical properties**

| **Amino acid** | **Factor 1** | **Factor 2** | **Factor 3** | **Factor 4** | **Factor 5** | **Factor 6** | **Factor 7** | **Factor 8** | **Factor 9** | **Factor 10** |
| --- | --- | --- | --- | --- | --- | --- | --- | --- | --- | --- |
| A | -1.56 | -1.67 | -0.97 | -0.27 | -0.93 | -0.78 | -0.2 | -0.08 | 0.21 | -0.48 |
| R | 0.22 | 1.27 | 1.37 | 1.87 | -1.7 | 0.46 | 0.92 | -0.39 | 0.23 | 0.93 |
| N | 1.14 | -0.07 | -0.12 | 0.81 | 0.18 | 0.37 | -0.09 | 1.23 | 1.1 | -1.73 |
| D | 0.58 | -0.22 | -1.58 | 0.81 | -0.92 | 0.15 | -1.52 | 0.47 | 0.76 | 0.7 |
| C | 0.12 | -0.89 | 0.45 | -1.05 | -0.71 | 2.41 | 1.52 | -0.69 | 1.13 | 1.1 |
| Q | -0.47 | 0.24 | 0.07 | 1.1 | 1.1 | 0.59 | 0.84 | -0.71 | -0.03 | -2.33 |
| E | -1.45 | 0.19 | -1.61 | 1.17 | -1.31 | 0.4 | 0.04 | 0.38 | -0.35 | -0.12 |
| G | 1.46 | -1.96 | -0.23 | -0.16 | 0.1 | -0.11 | 1.32 | 2.36 | -1.66 | 0.46 |
| H | -0.41 | 0.52 | -0.28 | 0.28 | 1.61 | 1.01 | -1.85 | 0.47 | 1.13 | 1.63 |
| I | -0.73 | -0.16 | 1.79 | -0.77 | -0.54 | 0.03 | -0.83 | 0.51 | 0.66 | -1.78 |
| L | -1.04 | 0 | -0.24 | -1.1 | -0.55 | -2.05 | 0.96 | -0.76 | 0.45 | 0.93 |
| K | -0.34 | 0.82 | -0.23 | 1.7 | 1.54 | -1.62 | 1.15 | -0.08 | -0.48 | 0.6 |
| M | -1.4 | 0.18 | -0.42 | -0.73 | 2 | 1.52 | 0.26 | 0.11 | -1.27 | 0.27 |
| F | -0.21 | 0.98 | -0.36 | -1.43 | 0.22 | -0.81 | 0.67 | 1.1 | 1.71 | -0.44 |
| P | 2.06 | -0.33 | -1.15 | -0.75 | 0.88 | -0.45 | 0.3 | -2.3 | 0.74 | -0.28 |
| S | 0.81 | -1.08 | 0.16 | 0.42 | -0.21 | -0.43 | -1.89 | -1.15 | -0.97 | -0.23 |
| T | 0.26 | -0.7 | 1.21 | 0.63 | -0.1 | 0.21 | 0.24 | -1.15 | -0.56 | 0.19 |
| W | 0.3 | 2.1 | -0.72 | -1.57 | -1.16 | 0.57 | -0.48 | -0.4 | -2.3 | -0.6 |
| Y | 1.38 | 1.48 | 0.8 | -0.56 | 0 | -0.68 | -0.31 | 1.03 | -0.05 | 0.53 |
| V | -0.74 | -0.71 | 2.04 | -0.4 | 0.5 | -0.81 | -1.07 | 0.06 | -0.46 | 0.65 |

*Note*: Factors 1–10 were physiochemical factors established from a factor analysis of 188 available physiochemical properties of the 20 natural amino acids. By preserving 10 factors that described 86% variance of the original physiochemical properties, PRF can encode the physiochemical properties of each amino acid using a 10-dimensional vector. PFR, property factor representation.
