# Supplementary material for: DeepNitro: Prediction of Protein Nitration and Nitrosylation Sites by Deep Learning
Source: Genomics Proteomics Bioinformatics. 2018 Sep 27;16(4):294–306. doi: 10.1016/j.gpb.2018.04.007 (PMC6205083; doi:10.1016/j.gpb.2018.04.007)
Supplement: Supplementary Table S5 [file mmc10.docx]

**Table S5**  **Detailed parameter setting used for the deep neural network model**

|  | **Tyrosine Nitration and S-Nitrosylation** | | |  | **Tryptophan Nitration** | | |
| --- | --- | --- | --- | --- | --- | --- | --- |
|  | **Dimension** | **Activation function** | **Dropout** |  | **Dimension** | **Activation function** | **Dropout** |
| Input layer | 2040 |  |  |  | 40 |  |  |
| Layer 1 | 800 | ReLU | 0.9 |  | 30 | ReLU |  |
| Layer 2 | 500 | ReLU | 0.5 |  | 20 | ReLU |  |
| Layer 3 | 400 | ReLU | 0.5 |  | 10 | ReLU |  |
| Layer 4 | 300 | ReLU |  |  |  |  |  |
| Layer 5 | 200 | ReLU |  |  |  |  |  |
| Layer 6 | 50 | ReLU |  |  |  |  |  |
| Output layer | 2 | SoftMax |  |  | 2 | SoftMax |  |

*Note:* In each neural network, the momentum and learning rate of the stochastic gradient descent algorithm are set as 0.9 and 0.001, respectively. To avoid overfitting, L1 and L2 regularization are introduced, and the weights of L1 and L2 regularization are both set as 10^-4^. For output layer, the negative log-likelihood function is used as loss function.
