# Supplementary material for: DeepNitro: Prediction of Protein Nitration and Nitrosylation Sites by Deep Learning
Source: Genomics Proteomics Bioinformatics. 2018 Sep 27;16(4):294–306. doi: 10.1016/j.gpb.2018.04.007 (PMC6205083; doi:10.1016/j.gpb.2018.04.007)
Supplement: Supplementary Table S6 [file mmc11.docx]

**Table S6 Parameters of the simple MLP classifier used for abstraction ability evaluation**

|  | **MLP classifier** | |
| --- | --- | --- |
|  | **Dimension** | **Activation function** |
| Input layer | [800, 500, 400, 300, 200, 50] |  |
| Layer 1 | 100 | ReLU |
| Layer 2 | 50 | ReLU |
| Output layer | 2 | SoftMax |

*Note*: In the MLP classifier, the learning rate of the stochastic gradient descent algorithm is set as 0.001. For output layer, the negative log-likelihood function is used as loss function. MLP, multilayer perceptron.
