# Supplementary material for: DeepNitro: Prediction of Protein Nitration and Nitrosylation Sites by Deep Learning
Source: Genomics Proteomics Bioinformatics. 2018 Sep 27;16(4):294–306. doi: 10.1016/j.gpb.2018.04.007 (PMC6205083; doi:10.1016/j.gpb.2018.04.007)
Supplement: Supplementary Table S7 [file mmc13.docx]

**Table S7 Prediction performance for tyrosine nitration, tryptophan nitration, and *S*-nitrosylation with three different thresholds**

| **Modification** | **Threshold** | **Cutoff** | **Specificity** | **Sensitivity** | **Accuracy** | **MCC** |
| --- | --- | --- | --- | --- | --- | --- |
| **Tyrosine nitration** | High | 0.420 | 0.950 | 0.177 | 0.849 | 0.172 |
|  | Medium | 0.295 | 0.900 | 0.291 | 0.820 | 0.195 |
|  | Low | 0.227 | 0.850 | 0.385 | 0.789 | 0.206 |
| **Tryptophan nitration** | High | 0.642 | 0.951 | 0.506 | 0.818 | 0.537 |
|  | Medium | 0.576 | 0.903 | 0.578 | 0.806 | 0.516 |
|  | Low | 0.492 | 0.852 | 0.673 | 0.798 | 0.521 |
| ***S*-nitrosylation** | High | 0.434 | 0.950 | 0.153 | 0.820 | 0.153 |
|  | Medium | 0.370 | 0.900 | 0.294 | 0.801 | 0.212 |
|  | Low | 0.317 | 0.850 | 0.400 | 0.777 | 0.236 |

*Note*: The prediction thresholds were selected from 10-fold cross-validation. Specifically, low, medium, and high threshold were selected under specificity values of 0.85, 0.90, and 0.95, respectively. MCC, Matthews correlation coefficient.
